# Resistance switching behavior of atomic layer deposited SrTiO_3_ film through possible formation of Sr_2_Ti_6_O_13_ or Sr_1_Ti_11_O_20_ phases

**DOI:** 10.1038/srep20550

**Published:** 2016-02-02

**Authors:** Woongkyu Lee, Sijung Yoo, Kyung Jean Yoon, In Won Yeu, Hye Jung Chang, Jung-Hae Choi, Susanne Hoffmann-Eifert, Rainer Waser, Cheol Seong Hwang

**Affiliations:** 1 Department of Materials Science and Engineering and Inter-university Semiconductor Research Center, Seoul National University, Seoul 151-744, Korea; 2 Advanced Analysis Center, Korea Institute of Science and Technology, Seoul 136-791, Korea; 3 Center for Electronic Materials, Korea Institute of Science and Technology, Seoul 136-791, Korea; 4 Peter Gruenberg Institute (PGI-7), Forschungszentrum Juelich GmbH, and Juelich-Aachen Research Alliance (JARA-FIT), Juelich, Germany

## Abstract

Identification of microstructural evolution of nanoscale conducting phase, such as conducting filament (CF), in many resistance switching (RS) devices is a crucial factor to unambiguously understand the electrical behaviours of the RS-based electronic devices. Among the diverse RS material systems, oxide-based redox system comprises the major category of these intriguing electronic devices, where the local, along both lateral and vertical directions of thin films, changes in oxygen chemistry has been suggested to be the main RS mechanism. However, there are systems which involve distinctive crystallographic phases as CF; the Magnéli phase in TiO_2_ is one of the very well-known examples. The current research reports the possible presence of distinctive local conducting phase in atomic layer deposited SrTiO_3_ RS thin film. The conducting phase was identified through extensive transmission electron microscopy studies, which indicated that oxygen-deficient Sr_2_Ti_6_O_13_ or Sr_1_Ti_11_O_20_ phase was presumably present mainly along the grain boundaries of SrTiO_3_ after the unipolar set switching in Pt/TiN/SrTiO_3_/Pt structure. A detailed electrical characterization revealed that the samples showed typical bipolar and complementary RS after the memory cell was unipolar reset.

Redox-based oxide resistance switch (RS) device can be considered as the main category of the intriguing and futuristic electronic memory device, which is called resistance switching random access memory (RRAM). As there are a large number of diverse oxide systems with diverse and complex redox reactions, the detailed electrical characteristics of oxide-based RRAM are very much dependent on the constituent materials, the electrodes and the fabrication processes^1^,^2^,^3^,^4^,^5^. With too much of such diversity, compared with rather narrowed-down material system for phase change memories, Ge-Sb-Te alloys, highly active researches on RRAMs are ongoing in both academia and industries. However, such diversity also created certain challenges in determining the best choice for RRAM commercialization. In general, the oxide-based RS materials can be classified into two groups – one that contains a distinctive crystallographic phase as the locally conducting phase and the other that does not contain such phase. A typical example of the first is TiO_2_, where Magnéli phase materials, which can be represented by a general formula of Ti_n_O_2n-1_ (n = 2, 3, 4…), forms the conducing filament (CF), and the unipolar resistance switching (URS) of TiO_2_ film can be well understood from the repeated (thermal) rupture and (field-driven) rejuvenation of the Magnéli CF’s connecting the top and bottom electrodes^6^. In bipolar RS (BRS) of TiO_2_, the redox-like reaction, mediated by the electric field-driven migration of oxygen (vacancies) within the Magnéli CF ruptured region, well explains the detailed electrical RS behaviours^7^. By contrast, the HfO_2_ represents the second group of oxide-based RS materials, where the material system lacks a distinctive crystallographic phase which can form the local CFs. In this case, the field-driven oxygen vacancy (V_O_)-mediated redox reaction well explains the observed BRS, which also explains the general difficulty in observing URS in these material systems^8^. Recent work by Wang *et al.* revealed that Ohmic-like conduction of low resistance state (LRS) in HfO_2_ RS cell involved semiconducting CFs, suggesting the lack of a distinctive second phase, which could be metallic conductivity in this system^9^. The possibility of fine tuning in conductivity through fabrication process as well as the electrical operation in these material systems, since they lack the distinctive conducting phase, makes them favourable. Nevertheless, random switching of these conducting phases, by adding or loosing point defects, can invoke other critical problems such as significant random telegraph noise issues.

Perovskite oxide thin films attracted a great deal of attention during past several decades not only for RRAM, but also for many other functional electronic devices, such as dynamic random access memory and ferroelectric memory devices^10^,^11^,^12^,^13^,^14^,^15^,^16^,^17^,^18^. It is quite notable that one of the pioneering reports in RRAM field was based on one of the perovskites, Cr-doped SrZrO_3_, in year 2000^19^. Another highly notable report on the application of perovskite in RRAM involved ultimate scalability using the SrTiO_3_ (STO) single crystal and conducting atomic force microscopy, where dislocations were proven to be the repeatable source of electrical on/off operations^1^,^20^. These reports induced a great deal of interests in the perovskite oxides as a feasible medium for reliable RRAM, and one of the authors’ group (Waser group) conducted extensive studies on (doped) STO thin film systems^21^,^22^,^23^,^24^,^25^,^26^,^27^. In those studies, the redox reactions in STO based on the defect chemistry of this material were unambiguously identified as the predominant RS mechanism. The spatial-temporal evolution of local redox reaction across the film thickness was studied in detail. Nonetheless, the possible involvement of conducting second phase in STO, as in the case of TiO_2_, has not been seriously considered, except that the dislocations in STO have been discussed as the possible origin of CF formation^28^,^29^,^30^. Considering the fact that the crystal structure of STO is actually composed of corner-shared TiO_6_ octahedra, the overall stoichiometry of this corner-shared TiO_6_ octahedral network is TiO_3_, and thus, the excess negative −2 charge of the network per TiO_3_ unit is compensated by the included Sr^2+^ ions. Therefore, it is reasonable to assume that the redox reaction is mediated by the reduction and oxidation of Ti ions in the octahedral network. It also has to be noted that the conduction band of STO has a significant contribution from the Ti3*d* orbitals. One may also consider that there could be conducting second phases involved if the defect concentration (V_O_ concentration) becomes higher than its stability range. In TiO_2_, ~0.1% of [V_O_] is the stability range, and locally a very high V_O_ concentration induced the local phase transition into the Magnéli phase, which could be facilitated by the Joule–heating-assisted migration of oxygen ions during the RS operation^31^,^32^,^33^. However, it can be anticipated that such reaction in STO would be more complicated due to the presence of Sr ions, which might be the reason why such conducting phase has not been clearly identified in STO yet. According to the phase diagram of STO, oxygen deficient STO^34^,^35^,^36^ is known to be a good semiconductor. For non-stoichiometric SrTiO_3_ (expected in grain boundary regions) phase separation and the formation of either Ruddlesden-Popper phases (Sr-rich)^37^,^38^ or Magnéli type phases (Ti-rich)^39^ were reported. Nevertheless, identifying the conducting second phase in any RS system is still of utmost importance for accurate and precise understanding of the RS mechanism. Therefore, the authors extensively examined the structure of STO RS cell after the URS set, which may have the highest possibility to observe the conducting second phase if there is any, using high-resolution transmission electron microscopy (HRTEM). In order to further improve the success rate of such trials, a highly reliable film deposition process, atomic layer deposition (ALD), was adopted which resulted in an extremely uniform and reliable electrical characteristics, facilitating this tough task affordable. In general, such HRTEM work suffers from the scarcity of possible CFs in the memory cell structure, so extensive and dedicated TEM studies were necessary to identify the Sr_2_Ti_6_O_13_ or Sr_1_Ti_11_O_20_ phases as the conducting second phase in this work. Another complication in this work was that these phases are almost always found from the grain boundary regions of the differently oriented STO grains. A grain boundary between STO grains with different orientations can lead to misconception on the presence of grain boundary phases by the Móire fringe effect, which is basically the interference of two differently oriented lattice fringes, so very careful examination of the observed second phase-like images must be performed.

After the careful examination of the ~70 HRTEM images showing the second phase-like images at the STO grain boundaries, monoclinic Sr_2_Ti_6_O_13_ or triclinic Sr_1_Ti_11_O_20_ phase, which has been discussed in previous works deeply or sketchily^40^,^41^,^42^,^43^,^44^,^45^,^46^,^47^,^48^,^49^,^50^, could be presumably identified. In the phase diagram for Sr-Ti-O system^51^, these phases are less well known. Since these phases can be described as Sr_2_Ti_6_O_(2+12−1)_ or Sr_1_Ti_11_O_(1+22−3)_ they could be the conducting second phases, although detailed electrical properties of them have not been reported yet. In order to confirm the electrical properties of these two Ti-rich second phases, theoretical calculations of their densities of states (DOSs) were performed using first principles. It can be further considered that these second phases themselves can have oxygen non-stoichiometry, i. e. Sr_2_Ti_6_O_(13±x)_ or Sr_1_Ti_11_O_(20±x)_, but this was not significantly taken into account in this work for the sake of simplicity. Furthermore, the electrical properties of the STO films, which were differently heat-treated to change the crystallographic structure, were also examined after the memory cell was URS reset. This can provide a detailed understanding on the possible redox reaction at the region where the presumable Sr_2_Ti_6_O_13_ or Sr_1_Ti_11_O_20_ phase CF’s are ruptured.

## Results

### Structural characterization of SrTiO_3_ film after unipolar set switching

**Figure 1:**
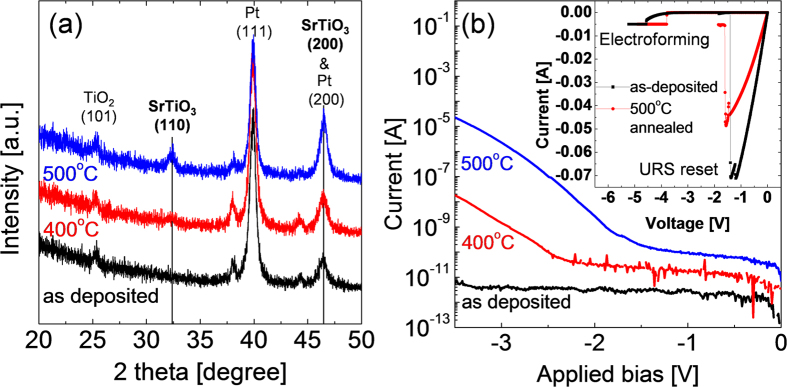
(**a**) Grazing angle incidence X-ray diffraction patterns, and (**b**) current versus applied voltage plots of as-deposited, 400 ^o^C annealed, and 500 ^o^C annealed STO films on Pt/TiO_2_/SiO_2_/Si substrate. TiN and Pt layers were deposited sequentially on STO layer for top electrode in (**b**). The inset figure in (**b**) shows the URS set (compliance current: 0.5mA) and reset switching curves of the as-deposited and 500 ^o^C annealed STO films.

Figure 1(a) shows the grazing angle incidence X-ray diffraction (XRD) spectra of the 12nm-thick STO film on 50nm-thick Pt/20nm-thick TiO_2_/300nm-thick SiO_2_/Si substrate. Pt is the bottom electrode (BE). Three kinds of STO films were prepared: the as-deposited state, partially crystallized state at 400 ^o^C, and practically crystallized film at 500 ^o^C by rapid thermal annealing (RTA) under N_2_ atmosphere for 2 min. The lack of crystalline diffraction peaks, except for those from the substrate materials, from the as-deposited state suggested that the STO film was amorphous, which is consistent with the fact that this ALD STO film was formed through a two-step growth process for *in-situ* crystallization. The TiO_2_ (101) peak at the as-deposited state was originated from the adhesion TiO_2_ layer beneath the Pt film. The RTA certainly crystallized the film into the perovskite structure as can be understood from the emergence of STO (110) peak near 2θ value of 32.5^o^ and increase in the peak intensity near 2θ value of 46.5^o^ which corresponded to STO (200) (and Pt (200)). Figure 1(b) shows the typical current – voltage (I-V) curves of the three kinds of STO films. The 50nm-thick Pt/5nm thick TiN (TiN contacts STO layer) top electrode (TE) was deposited by a dc sputtering through metal shadow mask (hole diameter 0.3mm). Due to the amorphous and dense microstructure of the as-deposited film, the current was very low down to −3.5 V; however, it increased with increasing RTA temperature due to crystallization-induced micro (nano) crack generation within the film^52^. Inset shows a typical electroforming and subsequent resetting I-V switching curves of the as-deposited film and the film ex-situ annealed at 500  ^o^C. Applying many different values of compliance current (I_cc_) was attempted to acquire repeatable URS switching behaviour, and 5 mA was identified to be an appropriate value. This enabled the stable URS set followed by the URS reset during voltage driven I-V sweep. In this sweep, the bias voltage was applied to the Pt/TiN TE while the Pt BE was grounded. The low current lines show electroforming behaviours, where the electroforming occurred at ~−4.6 and ~−3.8 V for the as-deposited STO and 500 ^o^C annealed STO films, respectively. The as-deposited STO film showed lower (absolutely higher) electroforming voltage than the 500 ^o^C annealed STO film due to its more insulating nature. Additionally, the high-current curve show reset curves, where the reset occurred at ~−1.5 V with a very high reset current of ~70 and ~50 mA for the as-deposited STO and 500 ^o^C annealed STO films, respectively. The detailed electroforming and subsequent reset/set parameters were subjected to rather large variations due to quite random nature of CF formation, rupture, and rejuvenation. This is a typical behaviour of oxide-based RRAM cell in the URS mode, especially when the distinctive conducting phase CF is involved. The URS behaviour of the as-deposited, and RTA films did not show any clear dependency (data not shown) on the degree of crystallization. The much higher reset current compared with the I_cc_ also suggested a high probability of the formation of a distinctive conducting phase as discrete CFs, which were further enhanced by the flow of stored charge in the high-k capacitor structure at the moment of URS set. It has to be noted that the semiconductor parameter analyser (SPA) adopted to perform the I-V sweep did not control the actual charge flow at the moment of set even though I_cc_ was fixed; it just decreased the voltage to a value at which the I_cc_ was detected. Although the electrode-area independencies of set- and reset-state currents were not confirmed, such a typical URS behaviour confirmed that the switching occurred locally.

**Figure 2:**
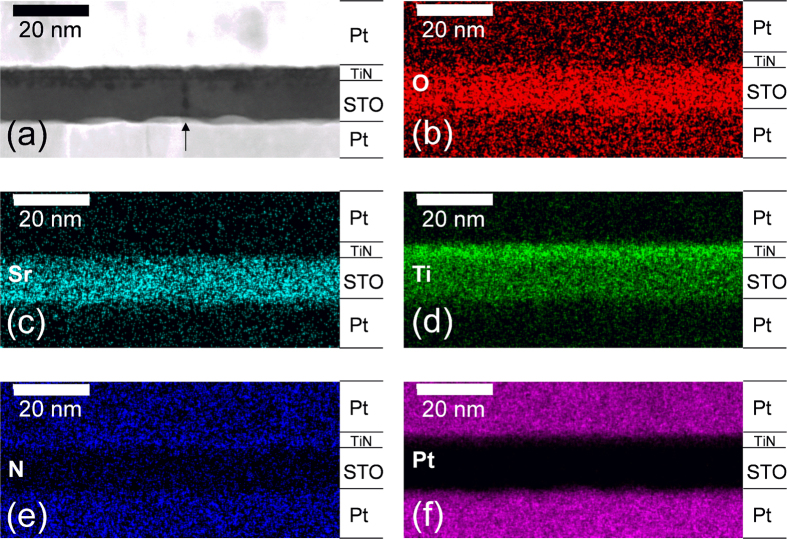
(**a**) Cross-section HAADFF image of Pt/TiN/(500 ^o^C annealed) STO/Pt structure which was electroformed with compliance current of 5 mA. The EDS mapping of (**b**) O, (**c**) Sr, (**d**) Ti, (**e**) N, and (**f**) Pt elements of the area where (**a**) was obtained.

URS electroforming and subsequent reset/set RS could be also induced by positive bias application to TE but the success rate was much lower as for the opposite bias case. This is probably due to the blocking effect of the intervened TiN layer on oxygen migration into the top Pt, whereas the same effect was not expected for the negative bias. This can be confirmed from the elemental mapping of the electroformed sample with negative bias, as shown in Fig. 2.

Figure 2(a) shows a high-angle annular dark field (HAADF) image in scanning TEM (STEM) of the cross-section of an electroformed (negative bias applied to TE), 500 ^o^C RTA sample. The picture was taken from an area where a grain boundary of the STO film was present, as indicated by the arrow in the figure. The STO film shows a very uniform thickness across the entire electrode area that could be observed during the TEM experiment, which is consistent with the general expectation for a uniform film growth in ALD. The average lateral grain size was ~50 nm. The interface region of STO in contact with TiN appeared to contain many dark spots in STEM indicating a certain chemical interaction of the STO with TiN either during the sample fabrication or electroforming. The RTA was performed before the TE deposition, so thermal diffusion of elements during the RTA was not the cause for the appearance of the dark spots. The grain boundary also showed a darker contrast in STEM, which indicated either lower density or loss of heavier elements in these regions in HAADF image. Figure 2(b)–(f) show O, Sr, Ti, N, and Pt elemental maps using energy dispersive spectroscopy (EDS). Due to the generation of spurious X-rays in such TEM analysis and diffuse scattering of electrons in the sample, spatial resolution of EDS mapping was limited. Nevertheless, there were several notable findings from these mapping results. First, oxygen EDS signal intensities in the top and bottom Pt layers were unexpectedly high, which might be related with the oxygen adsorption on the TEM sample surface. Moreover, the intensity was generally higher within the BE than within the TE, although the difference was not very large. This was consistent with the bias application direction of the electroforming process. During the electroforming process, the oxygen ions migrated from the STO layer into the BE according to the bias. Of course, high signal intensity of O in STO layer confirmed the presence of the STO layer. Furthermore, the Sr signal intensity near the interface region with TiN layer was locally low, although it was not very clear too, suggesting diffusion of some Sr into the TiN layer. This may have an important implication on the formation of Sr-deficient conducting second phase in this sample. The locally stronger signal of Ti than the bulk STO along the TE interface indicated that TiN layer remained at that location even after the electroforming. However, the noisy signal of N in EDS did not confirm whether it is TiN or TiO_x_N_y_ or even TiO_x_. The clearly confined Pt signals within the TE and BE regions rejected the possibility of any Pt migration into the dielectric film. The EDS mapping could not confirm what caused the dark contrast near the grain boundary indicated by the arrow in Fig. 2(a), due to the limited spatial resolution. Therefore, HRTEM was adopted and the sample was extensively examined. In this regard, HRTEM pictures of approximately 70 grain boundaries were taken and analysed by fast-Fourier transformation (FFT) and inverse FFT (iFFT) techniques to exemplify the presence of second phases. As mentioned previously, the possible misinterpretation of Móire fringe effect as the presence of second grain boundary phase was carefully taken care of, so the images that can be evidently regarded as the representation of the second phase were included. The detailed processes for excluding the diffraction spots by the multiple scattering and Móire fringe effect have been explained in on-line supplementary information (SI).

**Figure 3:**
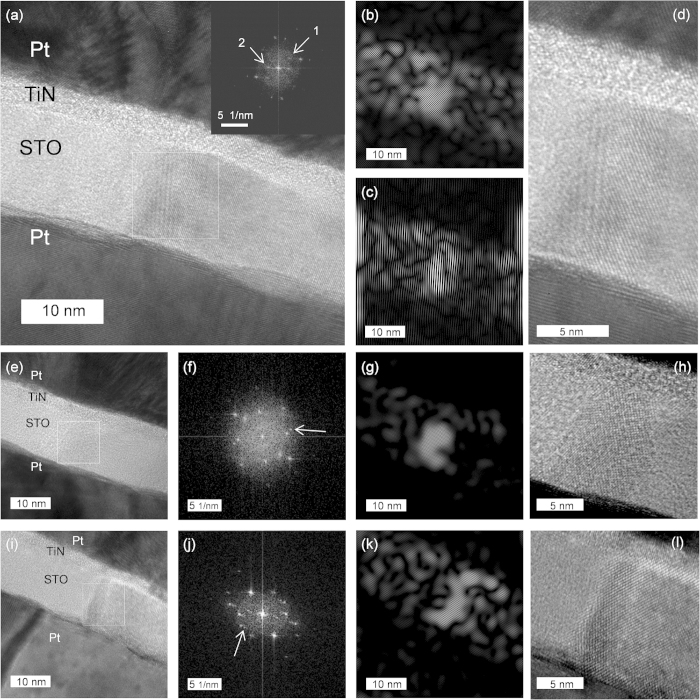
(**a,e,i**) Cross-sectional TEM images of electroformed Pt/TiN/(500 ^o^C annealed) STO/Pt and (inset of **a, f, j**) FFT patterns from the white boxes of (**a**), (**e**), (**i**). (**b,c,g,k**) iFFT images from the diffraction spots pointed by arrows in FFT patterns in (1, 2 of inset of **a**), (**f**), (**j**). (**d,h,l**) Enlarged TEM images of (**a**), (**e**), (**i**) around the white boxes.

Figure 3(a) shows a typical HRTEM image showing the presence of second phase at the grain boundary of the STO film, and the inset figure shows the FFT diffraction pattern of the region indicated by the square box. All the diffraction spots except 1 and 2 could be assigned as the perovskite STO phase. In order to confirm what phase may contribute to the 1 and 2 diffraction spots, the inter-planar spacing of the crystal planes that produced the two diffraction spots were measured. The scale of measurement was confirmed by the perovskite spots. Table 1 shows the measured inter-planar spacings from the FFT diffraction patterns and the possible candidate planes from various phases that can be found from the crystallography materials data composed of Sr, Ti, and O. Other than the perovskite phase, the possible phase that corresponded to the extra spots was either Sr_2_Ti_6_O_13_ or Sr_1_Ti_11_O_20_ phase. These phases are strongly Sr and oxygen deficient phases, as mentioned before, compared with the stoichiometric STO phase, and, thus, could be a good candidate for the CFs in this material. The spatial distribution of these possible phases can be identified from the iFFT of these spots, as shown in Fig. 3(b) for spot 1 and (c) for spot 2. It can be understood that the phases were mainly located near the grain boundary region of the STO film, but a non-negligible contribution from inside of the grains was also identified. Figure 3(d) shows a magnified image of the grain boundary region, showing that the second phase extended from the TE to the BE interface. Figure 3(e,i) show another regions where the grain boundaries contained the second phase, whose FFT and iFFT images (from the extra spots indicated by the arrows in the respective FFT images) are included in Fig. 3 (f,j) and (g,k), respectively. The iFFT images show that the second phase was more confined to only grain boundary region, especially in (g), compared with the other images. Figure 3(h show the enlarged HRTEM images of the second phase which again confirmed that they extended from the TE to the BE interface. These extensive HRTEM studies showed that the electroforming of the STO film induced the formation of conducting phases, which were presumably Sr_2_Ti_6_O_13_ or Sr_1_Ti_11_O_20_ phase. It is instructive to consider that the Sr-O binding energy is much stronger than that of Ti-O bonding, so the Sr-deficient region of STO, probably initiated by the reaction with TiN layer, induced the oxygen-deficient phases, which finally evolved into the Sr_2_Ti_6_O_13_ or Sr_1_Ti_11_O_20_ phase. Despite the extensive HRTEM work, further research is necessary to clearly identify the crystallographic and electronic structures of these phases, which might be possible by adopting nano-diffraction technique and energy-filtered electron energy loss spectroscopy, which have not been very successful, perhaps due to the overall matrix phase in TEM samples.

The identification of CF distribution along the film surface direction of the film has been accomplished by conducting atomic force microscopy (CAFM)^53^,^54^. This technique was also attempted in this work, but was not feasible as described below. In order to examine the (laterally) local electrical conduction mediated by the CFs, TE must be removed after the test cell was electroformed or switched to LRS. This has been usually done by peeling off the TE using a scotch tape. However, the presence of thin TiN between the Pt layer and STO film makes the adhesion between the TE and STO film stronger than that between the BE and STO film. Therefore, the tape test always resulted in peeling off the TE/STO stack from the Pt BE or TE/STO/BE stack from the TiO_2_/Si substrate, making the CAFM test unaffordable. Wet etching test of the TE material using aqua regia induced too much damage to the STO film. Therefore, an alternative test was performed using an extremely thin TE (3nm-thick Pt/3nm-thick TiN), because this would render sufficiently low lateral electrical conduction along the thin TE to not interfere with the CAFM test. This new sample had been biased with a very thin top electrode in an identical manner as shown in the inset figure of Fig. 1(b), but was not successfully electroformed because of too high lateral resistance of the thin top electrode. Nevertheless, the local electrical conduction was confirmed using CAFM on this thin TE sample, and it was found that current flow was concentrated at the grain boundary regions of the STO film from the 500 ^o^C annealed film (See Fig. S6 of on-line SI). As previously mentioned, the evolution of CFs in these oxide thin films is generally mediated by the Joule-heating assisted field-induced migration of the oxygen ions. Hence, the local current along the grain boundaries (or nano-cracks) in this film would make the change to form the CFs along the grain boundaries much higher than inside of the bulk grains. Therefore, Sr_2_Ti_6_O_13_ or Sr_1_Ti_11_O_20_ was mostly found at the grain boundary in Fig. 3.

**Figure 4:**
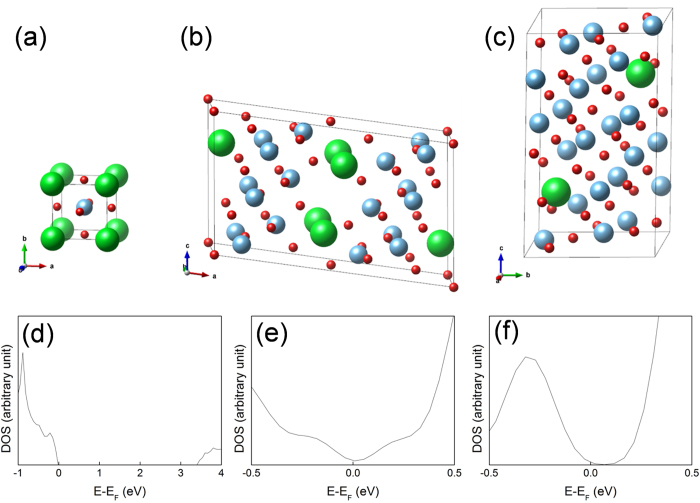
Crystal structures of (**a**) SrTiO_3_ (**b**) Sr_2_Ti_6_O_13_ and (**c**) SrTi_11_O_20_. Green, blue, and red circles denote Sr, Ti, and O atoms, respectively. Total density of states of (**d**) SrTiO_3_, (**e**) Sr_2_Ti_6_O_13_, and (**f**) SrTi_11_O_20_ which was obtained by HSE06.

While the structures of the Sr_2_Ti_6_O_13_ or Sr_1_Ti_11_O_20_ phases were reported^42^,^47^, detailed electrical properties of them are not fully understood. In order to complement this difficulty, the DOSs of the materials are theoretically calculated using the first principles. For SrTiO_3_, Sr_2_Ti_6_O_13_ and SrTi_11_O_20_, ab-initio calculations were performed using a pseudopotential plane-wave code known as the Vienna *ab-initio* Simulation Package (VASP)^55^,^56^. The projector augmented wave method within the generalized gradient approximation parameterized by Perdew *et al.*^57^, and HSE06^58^,^59^ were tested with a cutoff energy of 400 eV. The main calculations were performed using the HSE06, which showed the agreement on the bandgap of the experimental value of SrTiO_3_^60^. The 4s, 4p and 5s orbitals for Sr, 3p, 3d and 4s orbitals for Ti, and 2s and 2p orbitals for O were treated as the valence electrons. For the unit-cell of SrTiO_3_, Sr_2_Ti_6_O_13_, and SrTi_11_O_20_, the unit-cell volume and the atomic positions were fully relaxed until the Hellmann-Feynman force on each atom was reduced below 0.05 eV/Å. The 8 × 8 × 8, 2 × 8 × 4, and 4 × 4 × 2 Monkhorst-Pack k-point meshs^61^ were used to sample the Brillouin zone, respectively. To calculate the DOSs, finer k-points of 12 × 12 × 12, 4 × 16 × 8, and 8 × 8 × 4 were used in the Monkhorst-Pack scheme. Figure 4(a)–(c) represent the atomic structures of the SrTiO_3_, Sr_2_Ti_6_O_13_, and SrTi_11_O_20_, whereas Table 2 shows their optimized structures and band gaps in this study in comparison with the experimental values. Figure 4(d)–(f) display the total DOSs obtained by using HSE06 on SrTiO_3_, Sr_2_Ti_6_O_13_ and SrTi_11_O_20_. In contrast to SrTiO_3_, the two substoichiometric phase, Sr_2_Ti_6_O_13_ and SrTi_11_O_20_, show metallic characters (no band gap) indicating that they can act as conducting filaments, which correspond to the electrical tests results shown next.

**Figure 5:**
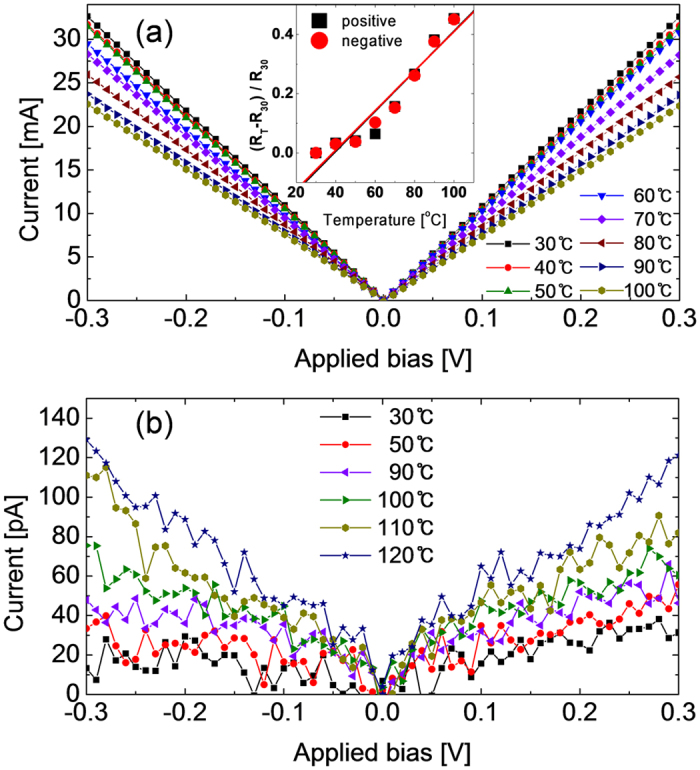
Current versus applied bias voltage plots of 500 ^o^C annealed STO films (**a**) after and (**b**) before electroforming at various measuring temperatures. The inset figure in (**a**) is the relative variation rate of resistance (reference temperature of 30 ^o^C) with measuring temperature.

Figure 5(a) show the I-V curves of the electroformed sample, shown in Fig. 3, at various measurement temperatures ranging from 30 to 100 ^o^C. The I-V curves show that Ohmic current flowed at all temperatures, and the curves were highly symmetric with respect to the bias polarity. The most notable finding was that the current decreased with increasing temperature. These results suggested that there are metallic conducting CFs connecting the TE and the BE in this case, which corresponds to the theoretical calculation results mentioned above. Due to the lack of precise information on the lateral distribution of these CFs across the electrode area, it was difficult to quantify the conductance of each CF, but it was quite obvious that the CFs, which were most probably Sr_2_Ti_6_O_13_ or Sr_1_Ti_11_O_20_ phase, were metallic. The inset figure shows the variation of relative resistance values with the reference at 30 ^o^C, estimated from the slopes of the I-V curves, as a function of temperature. The slope of the best-linear-fitted graph corresponded to the temperature coefficient of resistance, ~ 6.7 × 10^−3 o^C^−1^. This value is higher than that of Magnéli phase in TiO_2_ (~1.5 × 10^−5 o^C^−1^) by approximately two orders of magnitude, suggesting the more metallic properties of the Sr_2_Ti_6_O_13_ or Sr_1_Ti_11_O_20_ phase CFs in this work^65^.

Heavily reduced (degenerate semiconductor) SrTiO_3-x_, which was formed by thermal reduction at a temperature of 900 ^o^C, also showed similar temperature coefficient of resistance^66^. In contrast, the detailed electrical properties of Sr_2_Ti_6_O_13_ and Sr_1_Ti_11_O_20_ phases, including their temperature coefficient of resistance, have not been reported yet, although it is obvious that they have metallic conductivity^46^. Therefore, it is difficult to unambiguously identify the nature of CFs in this system presently. Nevertheless, Sr_2_Ti_6_O_13_ or Sr_1_Ti_11_O_20_ phase is suggested to be the main constituent of the CFs in this research as they are identified to be present across the entire film thickness at the electroformed state. By contrast, the I-V curves of pristine as-deposited STO film showed highly insulation behaviour at all temperatures, as shown in Fig. 5(b). Although the I-V curves were quite noisy, it was quite certain that the temperature dependency is insulating; current increased with increasing temperature. When it was reset by the I-V sweep, the current decreased abruptly (Fig. 1(b) inset), and it displayed insulating property at high resistance state (HRS), i.e. the current increased with increasing temperature. A more detailed discussion on the electrical properties of this URS reset state, which also corresponds to the BRS reset state, will be discussed in detail in the subsequent section.

### Bipolar resistance switching of URS reset memory cell

Next, the BRS behaviour of the Pt/TiN/STO/Pt memory cells in URS reset state was described. In this experiment, the BRS was always induced from the URS reset state, where the BRS set and the reset were generally observed under positive and negative bias polarities, respectively. As previously mentioned, electroforming with positive bias polarity has a low success rate, so attempt to directly achieve the BRS, including electroforming in positive bias polarity for BRS, without involving the previous URS was not successful. This strongly indicated that the BRS was induced at a local (in terms of both lateral and vertical directions) region where the presumable Sr_2_Ti_6_O_13_ or Sr_1_Ti_11_O_20_ phase CF’s were ruptured.

**Figure 6:**
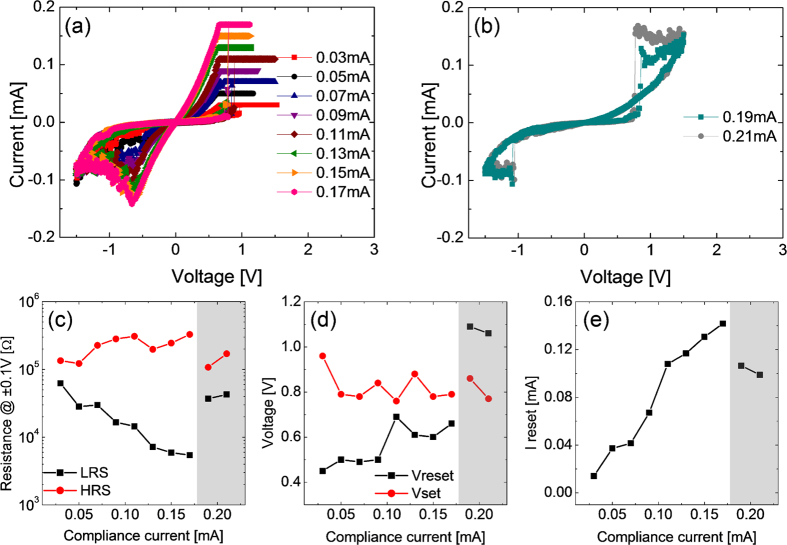
(**a**) BRS set and reset switching curves of as-deposited STO films with compliance current below 0.17 mA, and (**b**) CRS switching curves with compliance current of 0.19 and 0.21 mA. Variations of (**c**) resistance at ±0.1 V, (**d**) set and reset voltage, and (**e**) reset current as a function of compliance current of BRS set.

Figure 6(a) shows the BRS I-V curves with various I_cc_, from 0.03 mA to 0.17 mA, which was much smaller than the value for URS, during the BRS set in positive bias region of the as-deposited STO film. In the negative bias region, the film displayed a gradual decrease in current with further decrease in voltage after a certain voltage (V_reset_), which corresponded to the negative differential resistance (NDR) behavior. NDR behaviors have been commonly observed in many oxide-based RS cells during BRS reset^67^. By contrast, the BRS set occurred rather abruptly at a certain positive voltage (V_set_), which is rather uncommon in BRS set of oxide materials. This abrupt set shared a common feature with the URS, and such behaviour might have a close relationship with the appearance of complementary resistance switching (CRS) behavior at even higher I_cc_ values (0.19 and 0.21 mA), as shown in Fig. 6(b). The emergence of CRS was originally suggested for the two anti-parallel connected electrochemical metallization cells^68^, but such behavior has recently been reported in several single layer oxides^69^,^70^. Discussions on the emergence of CRS in this material system will be provided later. Figure 6(c–e) show the variations in the various BRS parameters as a function of I_cc_. Figure 6(c) shows that resistance of LRS (R_LRS_) decreased monotonically with increasing I_cc_, whereas the resistance of HRS (R_HRS_) was rather invariant, making the resistance ratio increase with increasing I_cc_. When the sample showed CRS, the R_LRS_ and R_HRS_ deviated quite significantly from the previous trends at lower I_cc_ values, which appeared reasonable when quite different I-V curves and switching mechanism were considered. In general, the resistance ratio of CRS with high I_cc_ was much smaller than that of BRS, when it was estimated at the same read voltage, ±0.1 V. However, it has been established that the reading of memory state in CRS requires a special voltage application procedure, so such simple comparison of resistance ratio does not hold significance. Absolute value of V_set_ was always higher than V_reset_, however, the trends for V_set_ and V_reset_ as a function of I_cc_ were not very obvious, and it might be reasonable to state that they were rather invariant with I_cc_ in the BRS region, but V_reset_ became abruptly very high when the sample started to show CRS behavior. The maximum current at the V_reset_ monotonically increased with increasing I_cc_ (Fig. 6(e)). This was reasonable because the higher I_cc_ during BRS set made the conduction channel, which was believed to be different from the CF in URS set state, more conducting.

**Figure 7:**
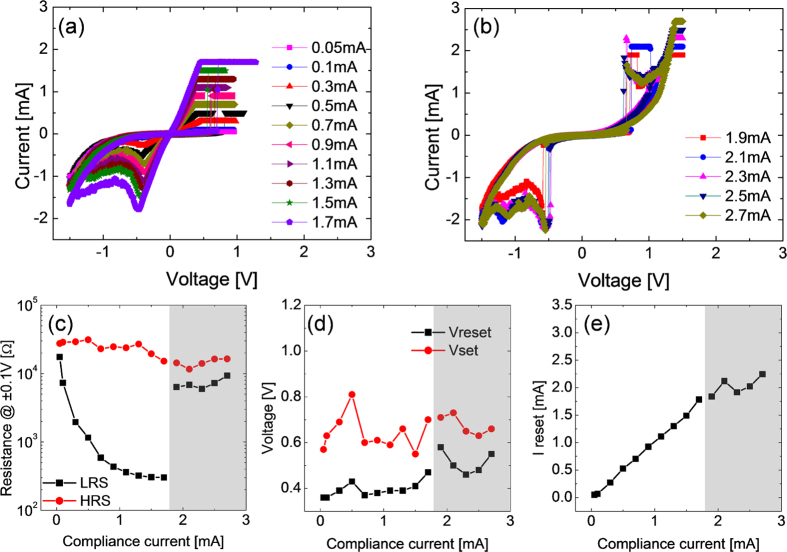
(**a**) BRS set and reset switching curves of 500^o^C annealed STO films with compliance current below 1.7 mA, and (**b**) CRS switching curves with compliance current over 1.9 mA. Variations of (**c**) Resistance at ±0.1V, (**d**) set and reset voltage, and (**e**) reset current as a function of compliance current of BRS set.

The crystallized STO film at 500 ^o^C showed slightly different BRS characteristics, as shown in Fig. 7. The film crystallized at 400 ^o^C showed similar BRS characteristics as the 500 ^o^C film, so the data are not shown. The crystallized STO film showed an identical BRS switching polarity as that of the as-deposited film (Fig. 7(a)), while the necessary I_cc_ level to observe the fluent BRS was certainly higher than the other case by almost one order of magnitude (0.05–1.7 mA). This was related to higher leakage current of the ex-situ crystallized film, as shown in Fig. 1(b), i.e. an integral part of the current was just consumed by the leakage current through the bulk region of the film. Accordingly, the R_LRS_ and R_HRS_ were generally lower than those of the amorphous film by approximately one order of magnitude (Fig. 7(c)), while the resistance ratio increased with increasing I_cc_ in this case too. This film also showed CRS behavior when I_cc_ became higher than 1.9 mA (Fig. 7(b)), which was also accompanied with a large decrease in the resistance ratio estimated at ±0.1 V. V_reset_ and V_set_ of this film also did not show any systematic variation with I_cc_, but they were generally lower than the other case. It is interesting to note that the V_reset_ and V_set_ of CRS were of similar magnitude with the BRS regime in this case, whereas it was not the case for the as-deposited film. I_reset_ showed an expectable trend, i.e. a linear increase with increasing I_cc_, and the general level of I_reset_ was higher than the other case, which was consistent with the higher leakage current of this film.

**Figure 8:**
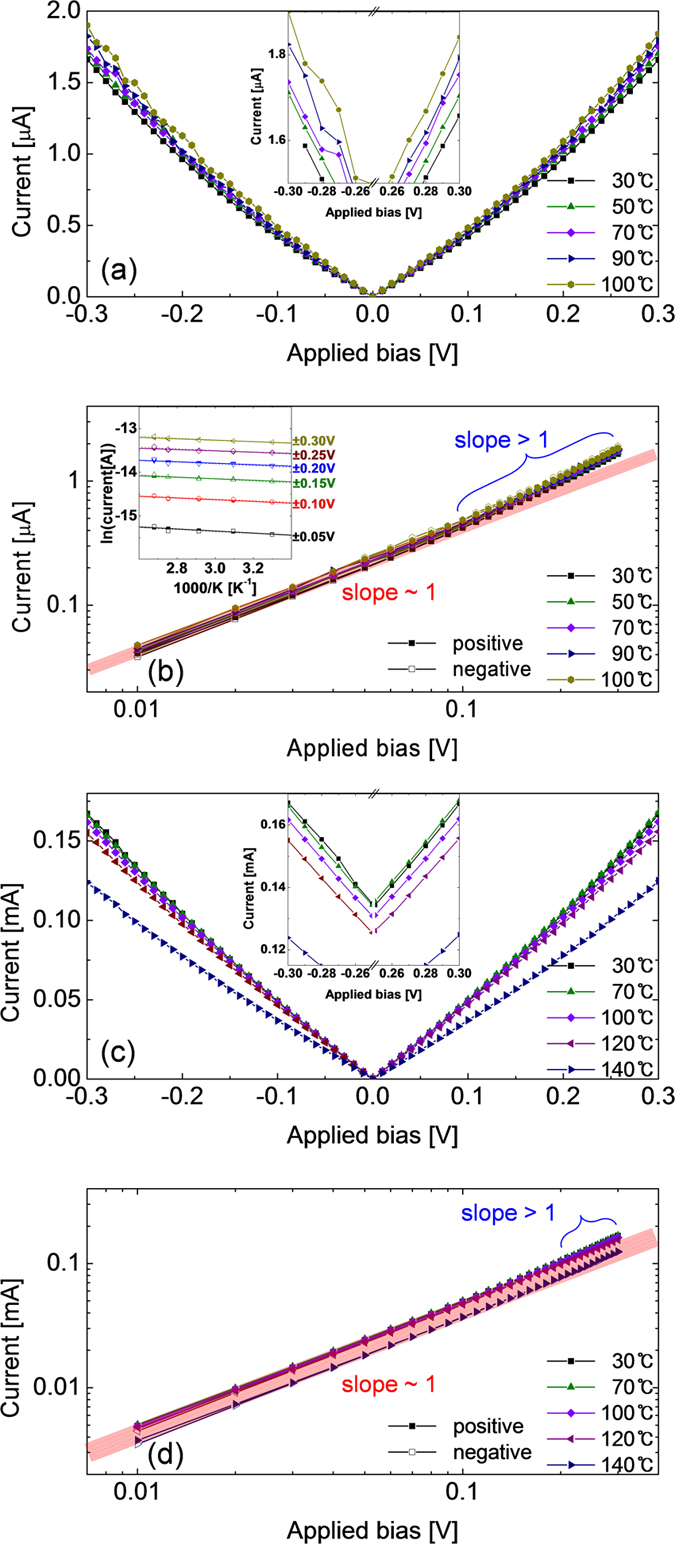
Current versus applied bias plots of STO films (**a**) after URS reset (also coincide with BRS reset) and (**c**) after BRS set. Log scale plots of the equivalent data (**b**) to URS reset and (**d**) to BRS set. Inset figures in (**a,c**) are the enlarged plots of (**a,c**) within ±0.3 V. Inset figure in (**b**) is the Arrhenius plot of current of STO films after URS reset. Numbers near the right axis indicate the voltages at which each current was estimated.

The temperature dependencies of the BRS reset and set states were also examined by measuring their I-V curves at various temperatures. For this experiment, the ex-situ crystallized film was BRS set with an I_cc_ of 0.5 mA and subsequently reset with a minimum reset voltage of −1.5V as in the case of Fig. 7(a). Figure 8(a) shows the I-V curves of the BRS reset sample, where the current was in the order of μA, suggesting that reset had been accomplished. The current increased with increasing temperature (Fig. 8(a) inset), suggesting the semiconducting nature of the conducting channel. It was certainly different from the highly insulating nature of the pristine state, suggesting that the local Sr_2_Ti_6_O_13_ or Sr_1_Ti_11_O_20_ phase CF ruptured region was responsible for the HRS in BRS. Figure 8(b) shows the logI - logV plot of the I-V curves, where almost Ohmic current flows (slope ~ 1) at absolute voltages < ~0.1 V, whereas a slightly higher slope was obtained at higher voltages. The activation energy of the current variation, estimated at <0.3 V was ~0.01−0.02 eV with a small variation (Fig. 8(b) inset). Such a small activation energy and Ohmic behavior suggested that the current flowed via hopping mechanism. This was consistent with the general view that the BRS reset region contains dispersed defects that mediate the hopping current flow, but the distance between them are long enough not to make a conducting channel^32^.

Figure 8(c) shows the I-V curves of the BRS set state sample at various temperatures. The current level was in the order of 0.1 mA, suggesting that the BRS set occurred. Interestingly, it also showed slight metallic behavior (inset figure), but in general the temperature trend was less obvious. In addition, the I-V data estimated at 140 ^o^C deviated largely from the trend showing the less conducting behavior. This suggested that the BRS set actually corresponded to the transient state in recovering the complete Sr_2_Ti_6_O_13_ or Sr_1_Ti_11_O_20_ phase CF within the CF ruptured region. It might be reasonable to assume that the metallic phase and semiconducting phases were intermixed and the metallic phases marginally maintained a connected form at this state. The quite high measurement temperature may disturb the weak connection of the meta-stable Sr_2_Ti_6_O_13_ or Sr_1_Ti_11_O_20_ phase network, and the current decreased abruptly, as shown in the figure. The logI – log V plot in Fig. 8(d) revealed that the Ohmic behavior was maintained up to a slightly higher voltage, which also coincided with the weak CF rupture by URS reset of this film (weak connection between remaining metallic phases within the active switching region in this case).

**Figure 9:**
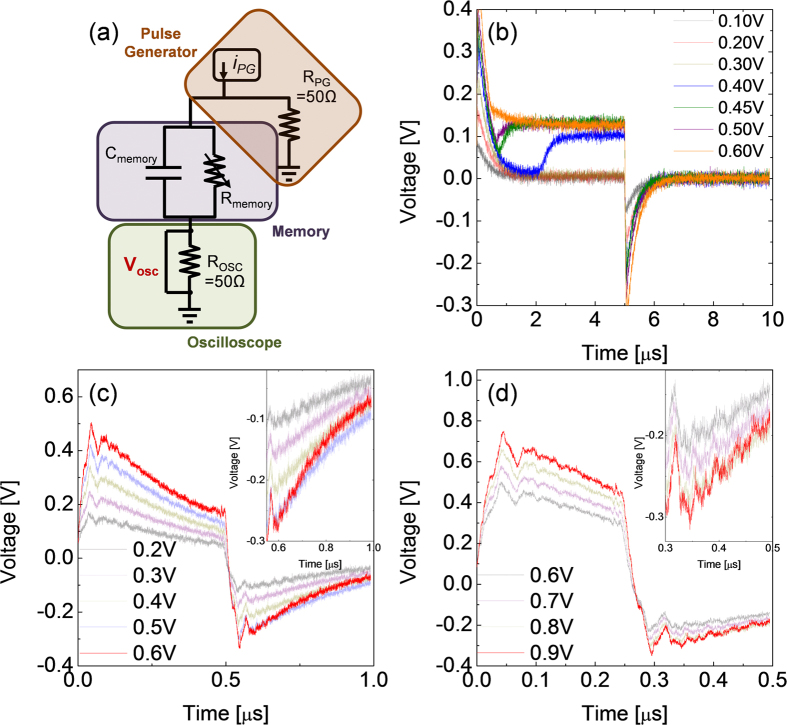
(**a**) Schematic diagram of the pulse switching circuit employed in this study. Voltage of oscilloscope versus time plots when voltage pulse of duration (**b**) 5μs, (**c**) 0.5μs, and (**d**) 0.25μs with various height were applied to 500 ^o^C annealed STO film. Inset figures of (**c,d**) are the enlarged plots of discharging peaks.

Finally, preliminary pulse switching (PS) experiments were performed on the ex-situ crystallized (500 ^o^C) film using the PS setup shown in Fig. 9(a). After the sample was electroformed and subsequently reset in the URS mode, the sample was pulsed with a 5μs-long positive pulses with different programmed amplitudes, in positive bias polarity, which was the BRS set polarity, as shown in Fig. 9(b). Due to the involvement of various parasitic components in the circuits as well as non-linearly varying sample resistance with voltage, the accurate estimation of the voltage applied to the sample in this setup was quite complicated, so only qualitative variation in oscilloscope voltage (V_OSC_) with time has been focussed here. Memory cell current, including the charging current component, can be easily monitored by dividing V_OSC_ with oscilloscope resistance (50 Ω). Figure 9(b) shows that there was hardly any change in the cell resistance for the programmed voltage up to 0.3 V, s o the sample showed simple capacitive charging (near time ~0s) and discharging (near time ~5μs) peaks. However, at the programmed voltage of 0.4 V, there was a sudden increase in V_OSC_ at ~2.5 s, which indicated that the sample was switched into the BRS set state. With further increase in the programmed voltage up to 0.5 V, the switching time shortened and at 0.6 V, the memory cell showed an almost instantaneous switching to LRS even before the capacitive charging ended. However, for all cases, significant discharging peaks were always observed even for the highest program voltage on termination of voltage pulse, suggesting that a substantial amount of charge remained stored even after the BRS set. This was quite a different situation from the URS set, where almost all the stored charges were drained off through the formed CF and no discharging peak appeared^71^. Yoon *et al.*^67^ reported that the conducting channel formed at the URS reset region by the BRS set had limited conductance, so similar discharging peak could be observed in TiO_2 _film. This meant that the formed conduction channel during the BRS set operation differed from the CFs formed by the URS set.

The dependence of the switching time on the program voltage was further examined in Fig. 9(c,d), where the voltage pulse duration was shortened to 0.5 μs and 0.25 μs, respectively. In these short periods of time, the evident change in V_OSC_, as in the case of Fig. 9(b), was not observed. However, the current decay rate after the termination of pulse was certainly different when BRS set occurred. In Fig. 9(c,d), the red curves for 0.6 V and 0.9 V, respectively, (inset shows enlarged portion) indicate that the delay time constant (RC time constant) was certainly smaller than the other cases, implying a decrease in resistance. The currents, measured at applied bias of 0.1V which is low enough not to induce any BRS, before and after the PS tests, indicated that the resistance was varied from ~1,500 to ~90 (Fig. 9(c)) and from ~1,600 to ~100 (Fig. 9(d)). These results revealed that the BRS switching time decreased with increasing pulse voltage, which was in accordance with the general trend in oxide RS materials. However, the present method cannot provide accurate information on the precise voltage – switching time relationship.

## Discussion

Similarity in BRS behavior and general emergence of CRS at higher I_cc_ values from the as-deposited amorphous and ex-situ crystallized STO films suggested that the RS in these materials occurs at several localized portions of the film, and the localized switching regions share quite similar properties in terms of RS. As previously mentioned, the BRS in these films was always triggered from the URS reset state, so discussion on the formation of CFs and their rupture in URS must be provided first. Oxide RS systems was composed of amorphous or crystalline RS layers, and polycrystalline electrodes, which inevitably encompass interfacial inhomogeneity, such as defects, local stresses, and protrusions. These heterogeneities induce localized current flow during the electroforming and subsequent RS steps. Such localized current can induce local Joule heating, so that even the amorphous film can be locally heated up to a quite high temperature which can crystallize that portion, in addition to the simultaneous formation of local CFs. It is believed that this is also the case for the ALD STO films in this work. The relatively open grain boundaries in ex-situ crystallized film might have provided local spots where current flowed mainly in the electroforming step, which then heated up that portion, thereby causing easy migration of oxygen atoms. (See Fig. S6 of on-line SI) This eventually formed the highly nonstoichiometric phases like the CFs, as elucidated in the previous section. For amorphous STO film, the localized current might have caused local crystallization of the film, and the boundary region between the crystalline phase and amorphous matrix could play the similar role as the grain boundaries in ex-situ crystallized film.

It is now widely accepted that the CFs in many oxide RS cells do not have uniform cross-sectional area and shape along their length direction. They could be either conical, dual conical or even hour-glass shaped^72^,^73^. Regardless of the shape, there is always a weaker part along their length direction, and the weaker part is responsible for the set and reset during repeated switching, whereas the rest stronger parts remained quite intact. In n-type oxide, such as TiO_2_, it has been reported that the CFs might have conical shape with the thicker part located near the cathode interface, so the weaker part near the anode interface was actually responsible for the switching (URS)^6^,^74^. A similar model can be also applied in the current work. When the Pt/TiN TE was negatively biased, oxygen ions in the film were pushed toward the BE Pt, and several local spots, mainly near the grain boundary or amorphous/crystalline interface, started to become devoid of sufficient oxygen. In this case, V_O_’s were generated at the STO/BE interface and were moved toward the TE interface. When sufficient number of V_O_’s accumulated at the STO/TiN interface, in conjunction with the Sr-deficient composition there because of the intermixing of STO and TiN, the film started to form highly non-stoichiometric Sr_2_Ti_6_O_13_ or Sr_1_Ti_11_O_20_ phase that extended toward the BE interface. Therefore, it is reasonable to assume that the Sr_2_Ti_6_O_13_ or Sr_1_Ti_11_O_20_ phase CFs had a conical shape where the thicker portion was in contact with the TiN TE, whereas the weaker portion made contact with Pt BE. It must be noted that TiN is generally much more impermeable to oxygen than Pt. Unfortunately, the HRTEM images (Fig. 3) did not reveal this geometrical shape clearly probably due to the involvement of many other interfering image effects. However, it was quite obvious that the CFs did not have a straight shape. Thus, it might be reasonable to assume that the Sr_2_Ti_6_O_13_ or Sr_1_Ti_11_O_20_ phase CFs in this work also had a conical (if not, at least not uniform diameter along the length) shape in general.

This model of the formation process of CFs in this asymmetric electrode system also elucidates the reason why electroforming with the positive bias to TE was not successful; under this circumstance, oxygen ions must penetrate through the TiN layer within the TE which was not very feasible compared with the opposite case. In addition, Sr-deficient region was near the TE interface, so formation of the non-stoichiometric Sr_2_Ti_6_O_13_ or Sr_1_Ti_11_O_20_ phase at the BE interface and their extension to the TE interface was highly improbable under this circumstance. For the case of symmetric Pt/TiO_2_/Pt case, the conical shaped CFs could be confirmed from the series-connected memory cell experiment^74^. However, the asymmetric electrode configuration, which was originally designed to impose the RS cell with appropriate asymmetry to induce BRS, did not allow such series connection experiment.

**Figure 10:**
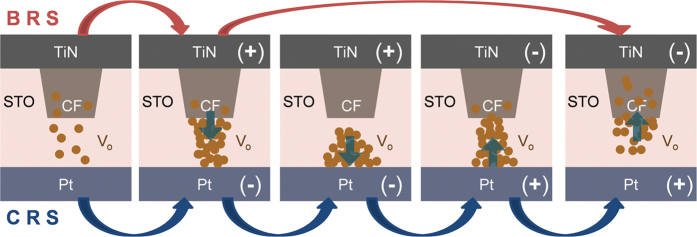
Schematic diagram of possible mechanisms for BRS and CRS switching in STO film. The left most image in this figure signifies post URS reset state.

It can be therefore assumed that during subsequent URS reset I-V sweep, the portion of CFs near the BE interface was ruptured while the stronger portion near TE interface remained rather intact, as shown in the left most diagram of Fig. 10. The CF ruptured region must contain relatively high V_O_ concentration, which was used to form the conducting channel during the subsequent BRS operation. It must be noted that the restriction of I_cc_ to the much lower value compared with the electroforming or URS set during the BRS set prohibited the full rejuvenation of Sr_2_Ti_6_O_13_ or Sr_1_Ti_11_O_20_ phase CFs. The operation of usual BRS (set in positive bias and reset in negative bias) can be readily explained by the repetition of the process indicated by the upper arrow in Fig. 10; the BRS set corresponded to the movement of some of V_O_ from the remaining Sr_2_Ti_6_O_13_ or Sr_1_Ti_11_O_20_ phase CFs into the CF ruptured region regaining conductance to a certain degree with the possible partial recovery of Sr_2_Ti_6_O_13_ or Sr_1_Ti_11_O_20_ phase, and the BRS reset corresponded to the recollection of these migrated V_O_’s back into the remaining CF. The emergence of CRS can be represented by the lower arrows in the figure. In this case, the high I_cc_ allowed further migration of V_O_’s toward the opposite interface after they connected the ruptured region temporarily. Such CRS can be induced only when the available density of V_O_’s was limited and sufficient driving force for their migration was available^75^. It is believed that the ordered crystalline structure of Sr_2_Ti_6_O_13_ or Sr_1_Ti_11_O_20_ phase CFs allowed only a certain amount of V_O_’s to move away from them during the BRS set (or CRS set), and when the driving force was sufficient, which was provided by the higher I_cc_, those limited amount of V_O_’s were swept across the CF ruptured region, as shown in the middle diagram of Fig. 10. When negative bias was applied to TiN TE, the oxygen vacancies migrated back to the CF and the reverse situation caused a high resistance state of device with intermediate low resistance state. It might be argued that the TEM images in Fig. 3 did not confirmed the conical shaped CF assumed in this discussion, but just a non-uniform CF configuration. As previously mentioned, many interfering effects in HRTEM observation, due to the confined configuration of CFs in STO matrix, prohibited the precise identification of the CF shape by TEM. Nevertheless, various electrical test results depending on the bias polarity supported that the conical shape of the CFs is a reasonable assumption.

In conclusion, the structure and electrical characteristics of RS memory cell composed of 11nm-thick amorphous or ex-situ crystallized STO films as the switching layer and Pt/TiN TE and Pt BE were examined. The memory cell showed fluent electroforming only when a negative bias was applied to TE, and subsequent BRS could be achieved with set and reset in positive and negative biases, respectively. Extensive and careful examination of the electroformed ex-situ crystallized sample through HRTEM and FFT technique revealed that the CFs, which were responsible for the metallic conductivity (increasing resistance with increasing temperature), were presumably composed of Sr_2_Ti_6_O_13_ or Sr_1_Ti_11_O_20_ phase. These phases were mainly present at the grain boundaries of the ex-situ crystallized film, and are supposed to be present at the boundary between the amorphous-matrix and local crystalline phases as well. They were formed by the local current flow and subsequent Joule heating in the as-deposited amorphous film. The fluent BRS was induced from the URS reset state of the STO film, both in the as-deposited and ex-situ crystallized films. It is believed that the local CF ruptured region near the Pt BE, which was due to the conical shape of the CFs with weaker part near the BE interface, partly recovered the electrical conductivity through V_O_ migration from the remaining CFs during the BRS set. BRS reset corresponded to the recollection of V_O_ into the remaining CF, suggesting that the remaining CF acted as a V_O_ reservoir. However, it was also found that the role of CF as the V_O_ reservoir was limited, so a large I_cc_ during BRS set induced CRS behaviour, which could be induced by the sweep of the aggregate of V_O_’s across the CF-ruptured region.

## Methods

A 50nm-thick Pt bottom electrode was prepared on sputtered 20nm TiO_2_/300nm SiO_2_/Si substrate by DC sputtering (Gmek Co.). STO films were deposited as RS layer in traveling-wave-type ALD reactor (CN-1 Co., Plus 100) with 4-inch-diameter scale. Sr(^i^Pr_3_Cp)_2_ (synthesized by Air Liquide Co.), (canister was heated to 90 ^o^C to obtain the appropriate vapour pressure) was employed as Sr-precursor for SrO deposition with the assistance of H_2_O (the canister was cooled to 5 ^o^C) as oxygen source. Here, iPr and Cp represent isopropyl and cyclopentadienyl ligands, respectively. Ti(CpMe_5_)(OMe)_3_ (synthesized by Air Liquide Co), (canister was heated to 80 ^o^C to obtain the appropriate vapour pressure), was used as Ti-precursor with high density (~250g/m^3^) O_3_ as oxygen source to deposit TiO_2_ layer. Here, CpMe and OMe represent methylcyclopentadienyl and methoxy ligands, respectively. Ar carrier gas flowed through the canisters of precursors at a flow rate of 200 standard cubic centimeters per min. and the process pressure was ~0.7 Torr. 3 s precursor injection, 5 s Ar purge, 2 s oxygen source injection, and 5 s Ar purge, which were confirmed as the saturated ALD condition, constructed one deposition cycle of SrO of TiO_2_^11^,^13^. One STO deposition cycle consisted of one TiO_2_ cycle and subsequent one SrO cycle for deposition of stoichiometric cation composition of STO films. A 12-nm-thick STO film was grown on Pt layer by 42 STO ALD cycles. Rapid thermal annealing (RTA) system maintained at 400 ^o^C or 500 ^o^C for 2min in the N_2_ atmosphere (purity > 99.99%) to produce STO films with a variety in crystallinity. With shadow mask (hole diameter 0.3mm), 5-nm-thick TiN was patterned on STO layer by reactive sputtering and 50 nm Pt was subsequently deposited on TiN by DC sputtering for stable probing. The consecutive layers of RS device were (top) Pt/TiN/STO/Pt (bottom) on TiO_2_/SiO_2_/Si substrate.

The layer density and cation composition of the STO films were confirmed by X-ray fluorescent spectroscopy (XRF, Themoscientific, ARL Quant’X). The physical thickness of the STO films was measured using an ellipsometer (Gaertner Scientific Corporation, L116D). The crystal structure of the film was investigated by grazing angle incidence X-ray diffraction, using a Cu Kα X-ray source (PANalytical, X’Pert Pro). The incidence angle, scan step size, and time per step during the GAXRD measurement were 2°, 0.02° and 1 s, respectively. The microstructure of the films was analysed using a high-resolution transmission electron microscopies (JEOL, JEM-2100F). The high-angle annular dark field images in scanning TEM and energy dispersive spectroscopy analyses were obtained by a second TEM (FEI, Talos F200X). The surface morphologies of films were confirmed by AFM (Jeol, JSPM-5200) and the localized conduction paths of STO film were investigated by CAFM (JEOL, JSPM-5200) measurement where the bottom electrode was positively biased and Pt-coated cantilever was grounded. The I-V curve was obtained by the semiconductor parameter analyser (HP 4145B) with conventional probe station. Pulse generator (HP 81110A) and oscilloscope (Tektronox 684C) were used for evaluation of electrical property in the case of pulse switching.

## Additional Information

**How to cite this article**: Lee, W. *et al.* Resistance switching behavior of atomic layer deposited SrTiO_3_ film through possible formation of Sr_2_Ti_6_O_13_ or Sr_1_Ti_11_O_20_ phases. *Sci. Rep.* **6**, 20550; doi: 10.1038/srep20550 (2016).

## Supplementary Material

Supplementary Information

## Figures and Tables

**Table 1:**
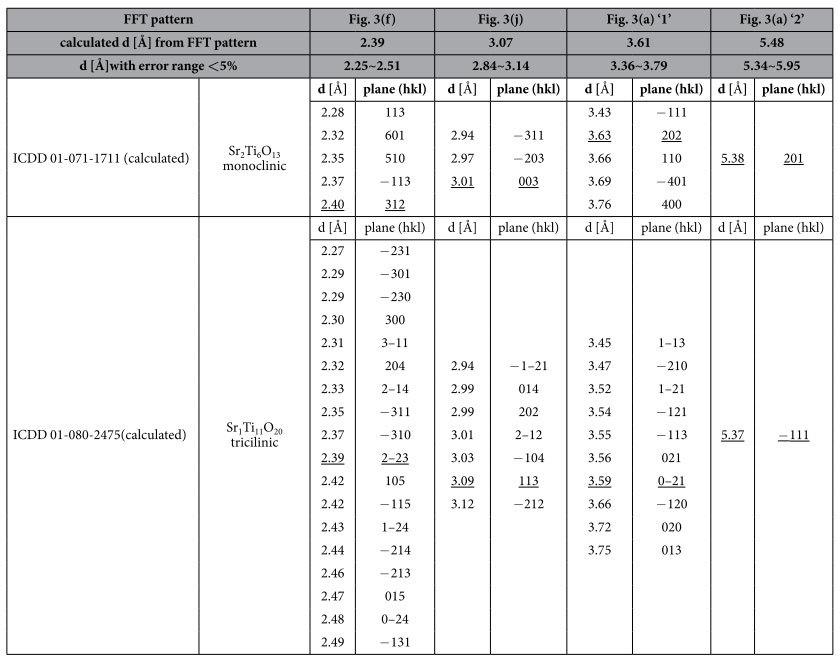
Calculated inter-planar spacings and the possible planes from the International Centre for Diffraction Data (ICDD) cards of Sr-Ti-O systems.

**Table 2:** Structural information on SrTiO_3_, Sr_2_Ti_6_O_13_, and SrTi_11_O_20_ and their band gaps.

	Space Group	XC- functional	Lattice Parameters a, b, c [Å]	α, β, γ [^o^]	Band gap [eV]	References
SrTiO_3_	Pm-3m	HSE06	3.88, 3.88, 3.88	90, 90, 90	3.35	This study
	cubic	experiment	3.90, 3.90, 3.90	90, 90, 90	3.25	60,62
Sr_2_Ti_6_O_13_	C2/m	HSE06	14.45, 3.85, 9.02	90, 97.4, 90	0	This study
	monoclinic	experiment	15.25, 3.77, 9.16	90, 99.2, 90	NA	63
SrTi_11_O_20_	P-1	HSE06	7.09, 7.63, 13.03	90.1, 93.1, 104.0	0	This study
	triclinic	experiment	7.13, 7.66, 13.16	90.2, 92.8, 103.9	NA	64
