# Supplementary material for: Resistance switching behavior of atomic layer deposited SrTiO3 film through possible formation of Sr2Ti6O13 or Sr1Ti11O20 phases
Source: Sci Rep. 2016 Feb 2;6:20550. doi: 10.1038/srep20550 (PMC4735854; doi:10.1038/srep20550)
Supplement: Supplementary Information [file srep20550-s1.pdf]

On-line Supplementary Information for

Resistance switching behavior of atomic layer deposited $SrTiO_3$ film through possible formation of $Sr_2Ti_6O_{13}$ or $Sr_1Ti_{11}O_{20}$ phases

Woongkyu Lee[1], Sijung Yoo[1], Kyung Jean Yoon[1], In Won Yeu[1,3], Hye Jung Chang[2], Jung-Hae Choi[3], Susanne Hoffmann-Eifert[4], Rainer Waser[4], and Cheol Seong Hwang[1,*]

[1]Department of Materials Science and Engineering and Inter-university Semiconductor Research Center, Seoul National University, Seoul 151-744, Korea
[2]Advanced Analysis Center, Korea Institute of Science and Technology,
Seoul 136-791, Korea
[3]Electronic Materials Research Center, Korea Institute of Science and Technology, Seoul 136-791, Korea
[4]Peter Gruenberg Institute (PGI-7), Forschungszentrum Juelich GmbH, and Juelich-Aachen Research Alliance (JARA-FIT), Juelich, Germany

[*]cheolsh@snu.ac.kr

Supplementary Information

Derivation of Moiré Equation and Verification of Diffraction Patterns in FFT Images from HRTEM

Clear identification of crystallographic second phases embedded in primary crystalline matrix phase by high-resolution transmission electron microscopy (HRTEM) is always challenging due to the overlapping lattice images, especially when the size of the second phase is of several nano-meter scale as was the case in this work. Under such circumstances, diffraction technique, either by the nano-beam diffraction or fast-Fourier transformation (FFT) of HRTEM images, could be helpful in identifying the crystallographic phases. However, even for the diffraction techniques, the overlap of diffraction patterns or spots from different phases is almost inevitable. Thus, clear distinction of the spots of the second phase from those of the matrix phase is very challenging. Additional factor that even augments the difficulty is the involvement of multiple scattering of electron that often produces Moiré patterns in images. In this work, as mentioned in the main text, the $Sr_2Ti_6O_{13}$ or $Sr_1Ti_{11}O_{20}$ phase were mostly found at the grain boundaries of the matrix $SrTiO_3$ phase, making the possible involvement of such side effects in imaging and diffraction analysis less reliable. Therefore, in this on-line Supplementary Information (SI), the method of how the extra-spots in the FFT patterns incurred by these side effects were identified was described in detail, which will reveal the accuracy and justification of the TEM analysis of Fig. 3 in the main text. More specifically, the method on how to analyze the extra-spots by the Moiré fringes originating from the difference in the lattice spacing of the involving phases and misfit in the orientation (rotation angle) of the given lattice fringes is described in detail in this SI.
Moiré pattern appears in most cases when two repeated patterns are superimposed with a small difference in lattice spacing or angles of the patterns. Several previous studies reported the equations for the cases where the angle and the periodicity are slightly different, but they were insufficient to describe all of the possible Moiré patterns that could have developed in this work.[1, 2] In this SI, the authors carefully followed the method of Gabrielyan[1] and the additional contemplation was complemented to achieve the general equation for Moiré pattern generation. In this SI, 'A' and 'B' represent the original patterns, whereas notation 'M' and 'm' are the Moiré pattern which could be originated by the interaction between the A and B patterns.
Gabrielyan derived the equation of the period of Moiré pattern (P_M) from two patterns with different periods along an identical direction.[1] As shown in Figure S1, new pattern is produced with a period of the distance between two perfectly overlapped positions. Colors of A(black) and B(grey) are selected only for better recognition and do not indicate the intensities of A and B patterns. They are assumed to have an identical intensity, but with disparate periods, P_A and P_B. When A and B are perfectly overlapped like A_0/B_0 and A_4/B_5, the white region around them becomes the largest, making that region bright, while the white region is mostly masked by A or B near $B_2/A_2/B_3$ making that region dark in the image. This overlap, therefore, can produce an additional pattern, which is represented by the sine-wave-like pattern at the bottom of Fig. S1 with a

new periodicity of P_M. Since a perfect overlapping can be observed when B has one more line than A does in a given distance (P_M), P_M can be derived from the P_A and P_B as in equation S1:

$$\frac{P_M}{P_B} = \frac{P_M}{P_A} + 1 \,, \quad P_M = \frac{P_A P_B}{P_A - P_B} \qquad \text{(S1)}$$

From equation (S1), Gabrielyan extended this concept to a somewhat general situlskation where the original patterns with different periods are located at a misfit angle between them.[1] Figure S2 shows two different patterns (A and B) with different periodicity (T_A and T_B) and angle from the X-axis (α_A and α_B) of a Cartesian coordinate. In this case, Moiré pattern (M) is developed with a period of T_M and angle of α_M from the X-axis. P_A and P_B are the Y-axis intercepts of A_I and B_I, respectively. The following two equations are deduced from Figure S2:

$$\tan\alpha_M = \frac{P_A + L\tan\alpha_A}{L} \qquad \text{(S2)}$$

$$\tan\alpha_B = \frac{P_A + L\tan\alpha_A - P_B}{L} \,, \quad L = \frac{P_A - P_B}{\tan\alpha_B - \tan\alpha_A} \qquad \text{(S3)}$$

, where L is denoted in the figure.

From equations (S2) and (S3), equation (S4) can be derived:

$$\tan\alpha_M = \frac{P_A + \dfrac{P_A - P_B}{\tan\alpha_B - \tan\alpha_A}\tan\alpha_A}{\dfrac{P_A - P_B}{\tan\alpha_B - \tan\alpha_A}} = \frac{P_A \tan\alpha_B - P_B \tan\alpha_A}{P_A - P_B} \qquad \text{(S4)}$$

In addition, the following equations (S5-1), (S5-2), and (S5-3) can be easily known from Figure 2S:

$$T_A = P_A \cos\alpha_A \qquad \text{(S5-1)}$$

$$T_B = P_B \cos\alpha_B \qquad \text{(S5-2)}$$

$$T_M = P_M \cos\alpha_M \qquad \text{(S5-3)}$$

From equations (S4), (S5-1), and (S5-2), equation (S6) which indicates the angle of Moiré pattern could be finally obtained:

$$\tan\alpha_M = \frac{T_A \sin\alpha_B - T_B \sin\alpha_A}{T_A \cos\alpha_B - T_B \cos\alpha_A} \,, \quad \boxed{\alpha_M = \tan^{-1}\left(\frac{T_A \sin\alpha_B - T_B \sin\alpha_A}{T_A \cos\alpha_B - T_B \sin\alpha_A}\right)} \qquad \text{(S6)}$$

Furthermore, the following formulas of trigonometry were used to derive T_M:

$$\cos\alpha = \frac{1}{\sqrt{1 + \tan^2\alpha}} \,, \quad \tan\alpha = \sqrt{\frac{1}{\cos^2\alpha} - 1} \qquad \text{(S7)}$$

$$\cos(\alpha_1 - \alpha_2) = \cos\alpha_1 \cos\alpha_2 + \sin\alpha_1 \sin\alpha_2 \qquad \text{(S8)}$$

From equations (S6), (S7), and (S8), the following equations can be deduced:

$$\frac{1}{\cos^2\alpha_M} - 1 = \left(\frac{T_A \sin\alpha_B + T_B \sin\alpha_A}{T_A \cos\alpha_B + T_B \cos\alpha_A}\right)^2 = \frac{T_A{}^2 \sin^2\alpha_B + T_B{}^2 \sin^2\alpha_A + 2T_A T_B \sin\alpha_B \sin\alpha_A}{T_A{}^2 \cos^2\alpha_B + T_B{}^2 \cos^2\alpha_A + 2T_A T_B \cos\alpha_B \cos\alpha_A}$$

$$\frac{1}{\cos^2\alpha_M} = \frac{T_A{}^2 + T_B{}^2 - 2T_A T_B(\sin\alpha_B \sin\alpha_A + \cos\alpha_B \cos\alpha_A)}{T_A{}^2 \cos^2\alpha_B + T_B{}^2 \cos^2\alpha_A - 2T_A T_B \cos\alpha_B \cos\alpha_A} = \frac{T_A{}^2 + T_B{}^2 - 2T_A T_B \cos(\alpha_B - \alpha_A)}{(T_A \cos\alpha_B - T_B \cos\alpha_A)^2}$$

$$\cos\alpha_M = \frac{T_A \cos\alpha_B - T_B \cos\alpha_A}{\sqrt{T_A{}^2 + T_B{}^2 - 2T_A T_B \cos(\alpha_B - \alpha_A)}} \qquad \text{(S9)}$$

Now, the equation (S5) becomes the following equation (S10) which is the period of Moiré fringe with equations (S1) and (S9):

$$T_M = \frac{T_A T_B}{T_A \cos\alpha_B - T_B \cos\alpha_A} \cdot \cos\alpha_M = \frac{T_A T_B}{T_A \cos\alpha_B - T_B \cos\alpha_A} \cdot \frac{T_A \cos\alpha_B - T_B \cos\alpha_A}{\sqrt{T_A{}^2 + T_B{}^2 + 2T_A T_B \cos(\alpha_B - \alpha_A)}}$$

$$\boxed{T_M = \frac{T_A T_B}{\sqrt{T_A{}^2 + T_B{}^2 - 2T_A T_B \cos(\alpha_B - \alpha_A)}}} \quad \text{(S10)}$$

Equation S10 coincides well with the Moiré fringe equation in another study.[2]

Meanwhile, Figure S3 shows another Moiré pattern which can be produced from the identical system of Figure S2. It should be considered that when $A_0/A_1/B_0/B_1$ (incarnadine parallelograms in Figures S2 and S3) is set to the unit parallelogram, the red line which connects the points A_0/B_0 and A_1/B_1 is not the only bright region in the developed Moiré pattern in this system. Although it cannot be easily recognized when α_B - α_A is small, the blue line which connects the points A_0/B_1 and A_1/B_0 could produce another Moiré pattern (m) which could be appeared as a new spot in FFT of the original image. Therefore, this second case should be also considered. In blue line, the equation (S1) must be slightly modified because it corresponds to the case with red lines in Fig. S2. For this new calculation, another parallelogram, $A_0/A_1/m_0/m_1$ (light blue parallelogram in Figures S3) was set as the basis for geometrical calculation where B_0 line corresponds to one of the diagonals of this new parallelogram. In this case, equation (S1) is modified as:

$$\frac{P_m}{P_B} = -\frac{P_m}{P_A} + 1 \quad , \quad P_m = \frac{P_A P_B}{P_A + P_B} \quad \text{(S11)}$$

Deriving the angle and the period of Moiré pattern, m, is analogous to that of the Moiré pattern M, except the adoption of equation (S11) instead of (S1). From Figure S3, equation (S12) and (S13) can be acquired.

$$\tan\alpha_m = \frac{P_A + l \tan\alpha_A}{l} \quad \text{(S12)}$$

$$\tan\alpha_B = \frac{P_A + l \tan\alpha_A + P_B}{l} \quad , \quad l = \frac{P_A + P_B}{\tan\alpha_B - \tan\alpha_A} \quad \text{(S13)}$$

Combining equations (S12) and (S13) results in the equation (S14):

$$\tan\alpha_m = \frac{P_A + \dfrac{P_A + P_B}{\tan\alpha_B - \tan\alpha_A}\tan\alpha_A}{\dfrac{P_A + P_B}{\tan\alpha_B - \tan\alpha_A}} = \frac{P_A \tan\alpha_B + P_B \tan\alpha_A}{P_A + P_B} \quad \text{(S14)}$$

From equations (S5) and (S14), the equation of the angle of Moiré fringe, m, from the X-axis can be deduced:

$$\tan\alpha_m = \frac{T_B \sin\alpha_A + T_A \sin\alpha_B}{T_B \cos\alpha_A + T_A \cos\alpha_B} \quad , \quad \boxed{\tan\alpha_m = \frac{T_B \sin\alpha_A + T_A \sin\alpha_B}{T_B \cos\alpha_A + T_A \cos\alpha_B}} \quad \text{(S15)}$$

With equations (S7), (S8), and (S15), following equations can be obtained:

$$\frac{1}{\cos^2\alpha_m} - 1 = \left(\frac{T_A \sin\alpha_B + T_B \sin\alpha_A}{T_A \cos\alpha_B + T_B \cos\alpha_A}\right)^2 = \frac{T_A{}^2 \sin^2\alpha_B + T_B{}^2 \sin^2\alpha_A + 2T_A T_B \sin\alpha_B \sin\alpha_A}{T_A{}^2 \cos^2\alpha_B + T_B{}^2 \cos^2\alpha_A + 2T_A T_B \cos\alpha_B \cos\alpha_A}$$

$$\frac{1}{\cos^2\alpha_m} = \frac{T_A{}^2 + T_B{}^2 + 2T_A T_B(\sin\alpha_B \sin\alpha_A + \cos\alpha_B \cos\alpha_A)}{T_A{}^2 \cos^2\alpha_B + T_B{}^2 \cos^2\alpha_A + 2T_A T_B \cos\alpha_B \cos\alpha_A} = \frac{T_A{}^2 + T_B{}^2 + 2T_A T_B \cos(\alpha_B - \alpha_A)}{(T_A \cos\alpha_B + T_B \cos\alpha_A)^2}$$

$$\cos\alpha_m = \frac{T_A \cos\alpha_B + T_B \cos\alpha_A}{\sqrt{T_A{}^2 + T_B{}^2 + 2T_A T_B \cos(\alpha_B - \alpha_A)}} \quad \text{(S16)}$$

Also, equation (S17) can be obtained from Figure S3:

$$T_m = P_m \cos\alpha_m \quad \text{(S17)}$$

Consequently, the period of Moiré fringe m can be obtained by equations (S11), (S16), and (S17):

$$T_m = \frac{T_A T_B}{T_A \cos\alpha_B + T_B \cos\alpha_A} \cdot \cos\alpha_m = \frac{T_A T_B}{T_A \cos\alpha_B + T_B \cos\alpha_A} \cdot \frac{T_A \cos\alpha_B + T_B \cos\alpha_A}{\sqrt{T_A{}^2 + T_B{}^2 + 2T_A T_B \cos(\alpha_B - \alpha_A)}}$$

$$T_m = \frac{T_A T_B}{\sqrt{T_A^2 + T_B^2 + 2T_A T_B \cos(\alpha_B - \alpha_A)}} \quad \text{(S18)}$$

Since the equations regarding Moiré pattern, m, are not very well-known, equation (S18) was also derived in another way by the cosine law. Figure S4 shows the incarnadine parallelogram which is identical to the incarnadine parallelograms in Figures S2 and S3. AA and BB are the lengths of A_0/B_0~A_0/B_1 and A_0/B_0~A_1/B_0 and MM is the length of the blue diagonal line. From Figure S4, AA and BB can be obtained:

$$AA = \frac{T_B}{\cos(90 - \alpha_B + \alpha_A)} = \frac{T_B}{\sin(\alpha_B - \alpha_A)} \quad \text{(S19-1)}$$

$$BB = \frac{T_A}{\cos(90 - \alpha_B + \alpha_A)} = \frac{T_A}{\sin(\alpha_B - \alpha_A)} \quad \text{(S19-2)}$$

Adopting the cosine law, MM can be expressed as following equation with aids of equations (S19-1) and (S19-2):

$$MM = \sqrt{AA^2 + BB^2 - 2AABB\cos(180 - \alpha_B + \alpha_A)} = \sqrt{AA^2 + BB^2 + 2AABB\cos(\alpha_B - \alpha_A)}$$
$$= \frac{\sqrt{T_A^2 + T_B^2 + 2T_A T_B \cos(\alpha_B - \alpha_A)}}{\sin(\alpha_B - \alpha_A)} \quad \text{(S20)}$$

When the area of the incarnadine parallelogram is considered, the following equation is derived.

$$2 \times \frac{1}{2} MMT_m = AABB\sin(\alpha_B - \alpha_A)$$

$$T_m = \frac{AABB\sin(\alpha_B - \alpha_A)}{MM} = \frac{\frac{T_A}{\sin(\alpha_B - \alpha_A)}\frac{T_B}{\sin(\alpha_B - \alpha_A)}\sin^2(\alpha_B - \alpha_A)}{\sqrt{T_A^2 + T_B^2 + 2T_A T_B \cos(\alpha_B - \alpha_A)}}$$

$$T_m = \frac{T_A T_B}{\sqrt{T_A^2 + T_B^2 + 2T_A T_B \cos(\alpha_B - \alpha_A)}} \quad \text{(S21)}$$

It is obvious that equation (S21) is equal to equation (S18) and the derivation of equations of Moiré pattern m for angle and period was confirmed. For the general equations of angle and period of Moiré patterns, equations (S6) and (S15) can be combined as following equations (S22) and (S23), respectively:

$$\alpha_{Moire} = \tan^{-1}\left(\frac{T_A \sin\alpha_B \pm T_B \sin\alpha_A}{T_A \cos\alpha_B \pm T_B \sin\alpha_A}\right) \quad \text{(S22)}$$

$$T_{Moire} = \frac{T_A T_B}{\sqrt{T_A^2 + T_B^2 \pm 2T_A T_B \cos(\alpha_B - \alpha_A)}} \quad \text{(S23)}$$

Next describes how such calculation can be utilized in ruling out the extra-spots due to the Moiré patterns in FFT images of the sample mentioned in the main text. Figures S5 (a)-(c) are the FFT images included as the inset in Figure 3 (a), Figure 3 (f), and (j), respectively. The diffraction spots that do not correspond to the inter-planar spacing value of $SrTiO_3$ from crystallography materials data are indicated by arrows on the right side of the images, and their corresponding inter-planar spacing and angle from the horizontal direction of the image (arbitrary reference, represented by the X-axis in Figs. S2-4) are also appended. To examine if the arrow-marked spot is originated from the actual second phase, such as the $Sr_2Ti_6O_{13}$ or $Sr_1Ti_{11}O_{20}$ phase, or due to the Moiré effect, combinations of every two spots were selected, and the angles and the periods of possible Moiré patterns were calculated using the equations (S22) and (S23), respectively. The physical dimensions of the reciprocal space were calibrated by the indexed diffraction spots from the $SrTiO_3$ phase. Table S1 shows the calculation results of all the possible Moiré patterns from the combinations of any two spots in the patterns in Figure S5, and produced patterns which are similar to the arrow-marked spots were shaded. Here, the base pattern corresponds to A and B patterns in Figs. S2-4. From these extensive simulations and comparisons to the experimental results, the diffraction spots which are marked with red arrows are turned out to be spots from the Moiré pattern while those marked with white arrows cannot be reproduced from any combination of the diffraction spots, suggesting that they are from the genuine second phases.

Distribution of Electrical Conduction of $SrTiO_3$ Film Analyzed by CAFM

The identification of planar distribution in electrical conduction along the surface direction was attempted via conducting atomic force microscopy (CAFM). For CAFM analysis, bias of 1 V was applied to the bottom electrode (Pt) and the AFM tip was grounded. CAFM study could have been performed on the electroformed device after removing the top electrode, but it was not feasible as described in the main text. Therefore, additional samples were fabricated as described below. Due to $SrTiO_3$ films' high insulating property for CAFM analysis, the current level obtained by the AFM tip with a very small contact point (~10 nm diameter) was as low as the noise level of equipment. This problem could be further degraded by high contact resistance between the CAFM tip and $SrTiO_3$ film surface. Accordingly, the 500ºC annealed $SrTiO_3$ film was coated with very thin top electrode (3 nm Pt / 3 nm TiN). This thickness was thin enough to interrupt electrical conduction along the lateral direction, while it substantially decreased the contact resistance and enabled the CAFM measurement. Figure S6 (a) shows the AFM topographic image of the 3 nm Pt/3 nm TiN/500ºC annealed STO sample and Figure S6 (b) displays the CAFM current image from the identical region of Fig. S6 (a). The new sample had been biased by utilizing very thin top electrode in an identical manner as shown in inset figure of Fig. 1 (b) before CAFM analysis. However, it was not successfully electroformed owing to the lateral resistance of the thin top electrode being too high. Nonetheless, the current mainly flowed near the grain boundary of $SrTiO_3$ as it can be seen in the Fig. S6 (c). Fig. S6 (c) is the overlapped figure of Figs. S6 (a) and (b). This indicates that Joule heating would be also concentrated at the grain boundary region, which is the prerequisite to migrate oxygen ions and to form the conducting second phases. Consequently, the conducting filaments must be generated at the grain boundary regions as it is consistent with the TEM observation in the main text. To confirm that the deposition of ultrathin top electrode did not change the grain size and shape of $SrTiO_3$ film in AFM topology image, the surface image of $SrTiO_3$ film without top electrode was also acquired and this is shown in Fig. S6 (d). The surface morphology was almost equivalent and grain sizes were ~ 50 nm before and after the top electrode deposition; it is consistent with the TEM results.

References

1. Gabrielyan, E., The basics of line Moiré patterns and optical speedup (2007) Available at: http://arxiv.org/abs/physics/0703098. (Accessed: 8th June 2015)

2. Williams, D. B. and Carter, C. B. Transmission Electron Microscopy - A Textbook for Materials Science 444-445 (Plenum Pess, 1996)

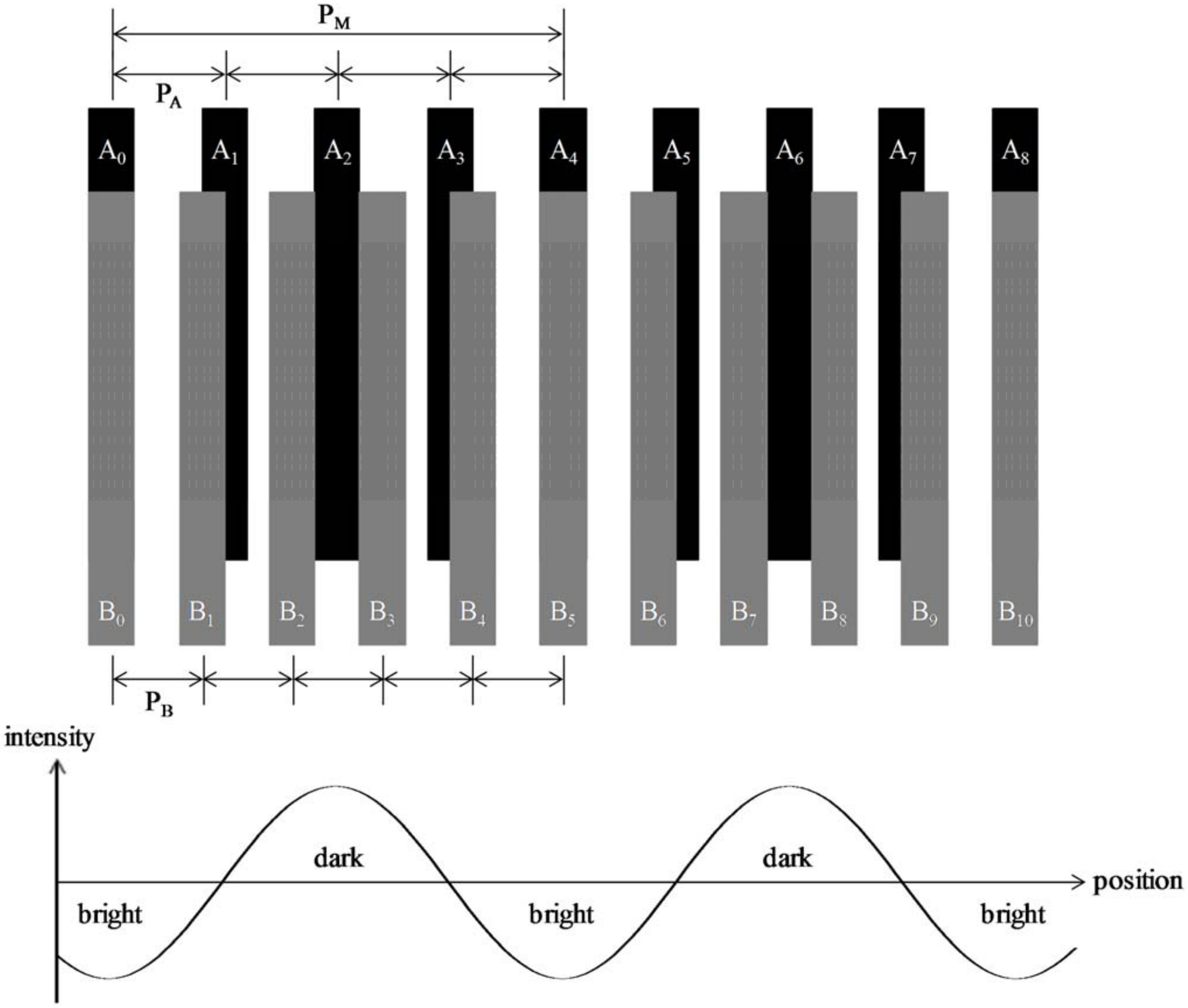

Figure S1. Superimposition of two patterns A and B with periodicity of P_a and P_b, respectively, in equivalent direction. Sine-wave-like Moiré pattern was newly developed.

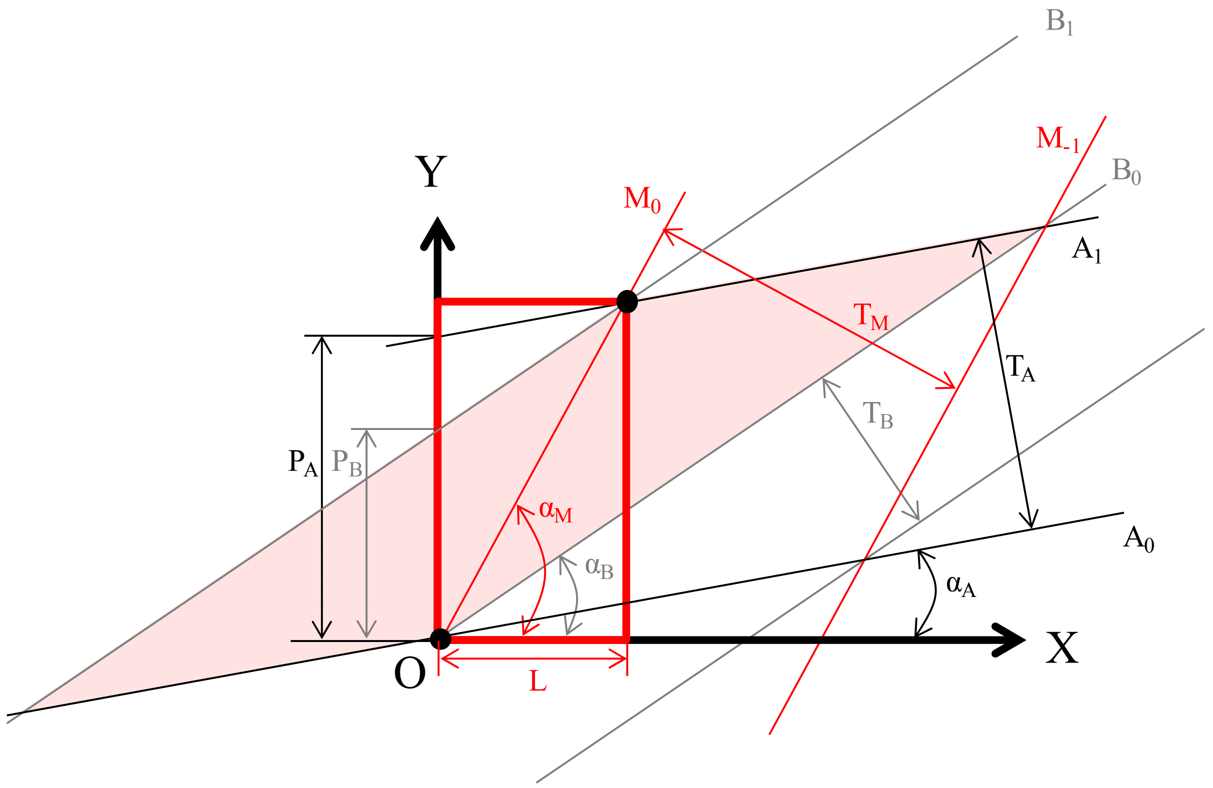

Figure S2. Two different patterns (A and B) with different period (T_A and T_B) and different angle from the X-axis (α_A and α_B). One Moiré pattern M (period: T_M, angle from the X-axis: α_M) was developed by A and B.

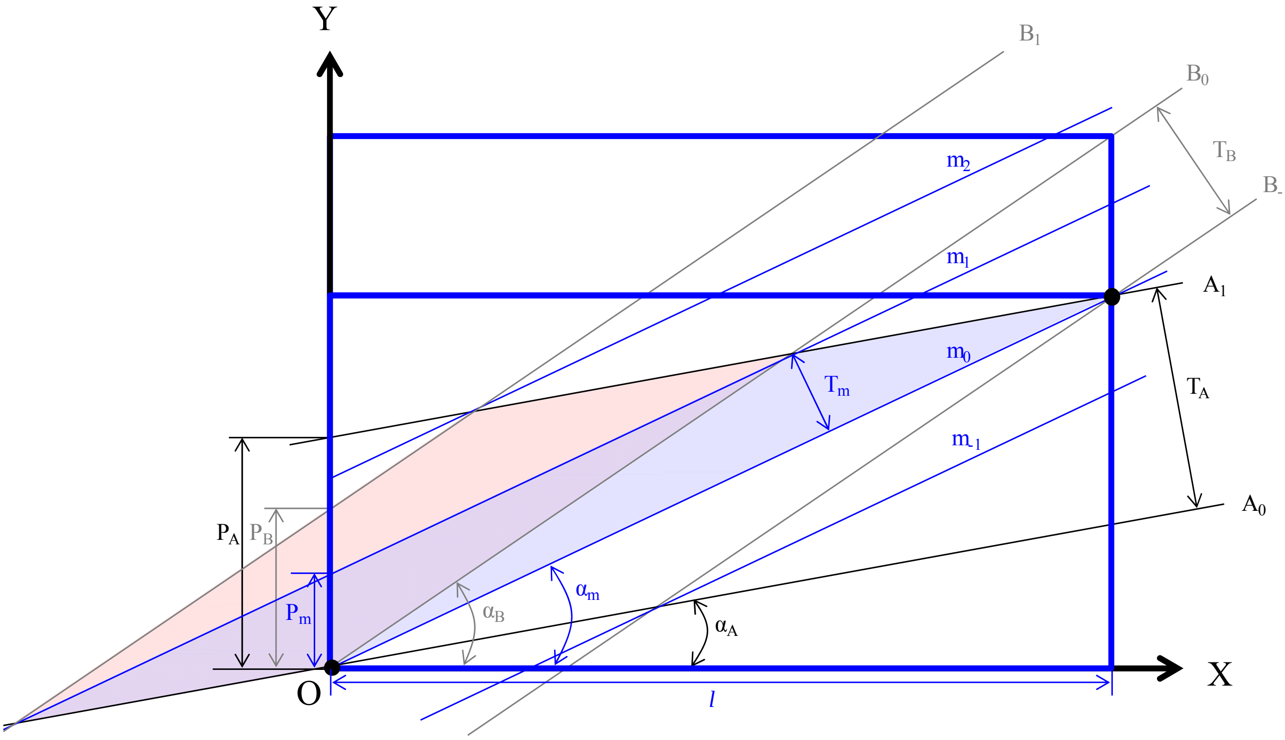

Figure S3. Two different patterns (A and B) with different period (T_A and T_B) and different angle from the X-axis (α_A and α_B). Another Moiré pattern m (period: T_m, angle from the X-axis: α_m) distinct from Moiré pattern M was also developed by A and B.

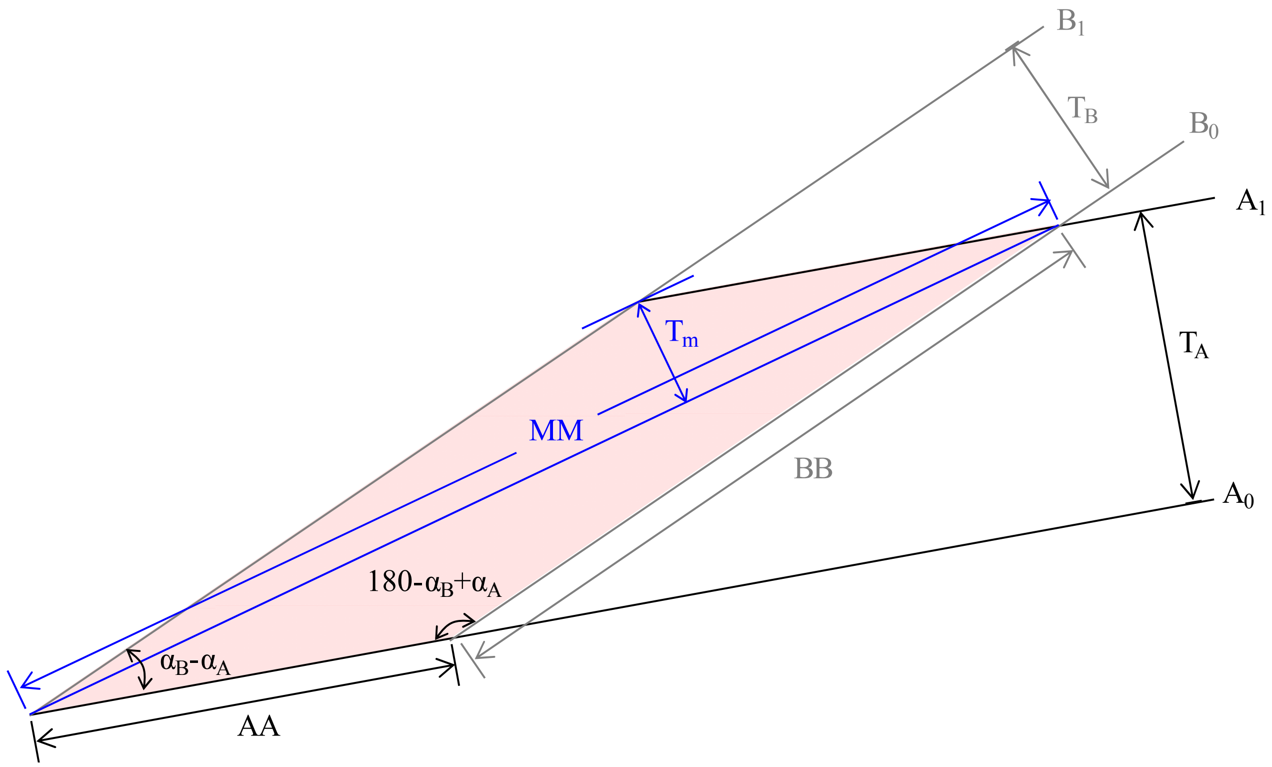

Figure S4. The equivalent incarnadine parallelogram in figure S2 and in figure S3 in distinguished view.

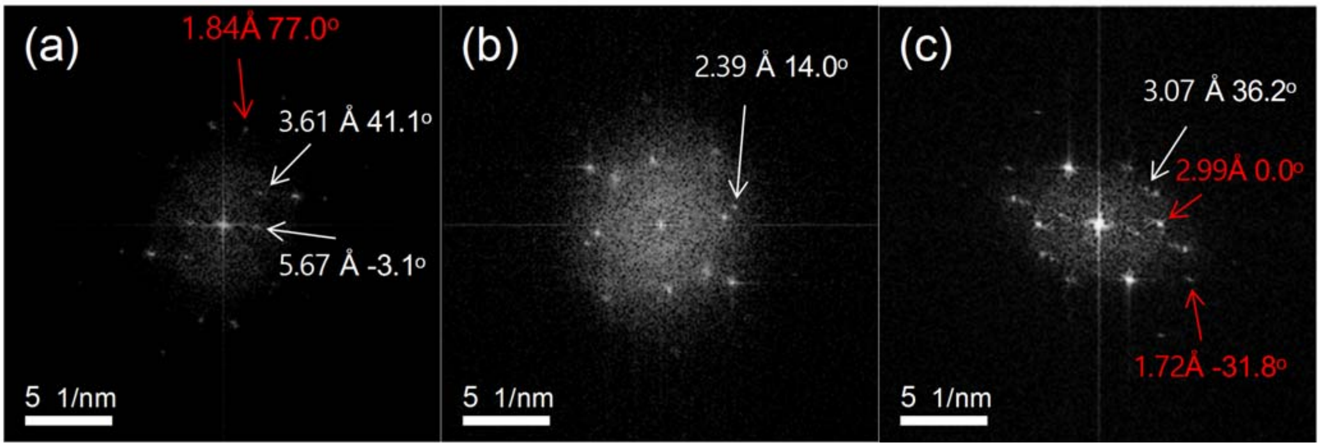

Figure S5. The FFT images equivalent to **(a)** the inset figure in Figure 3 (a), **(b)** Figure 3 (f), and **(c)** Figure 3 (j). The inter-planar spacings and angles from the horizon of the arrow-marked spots were included in the figures.

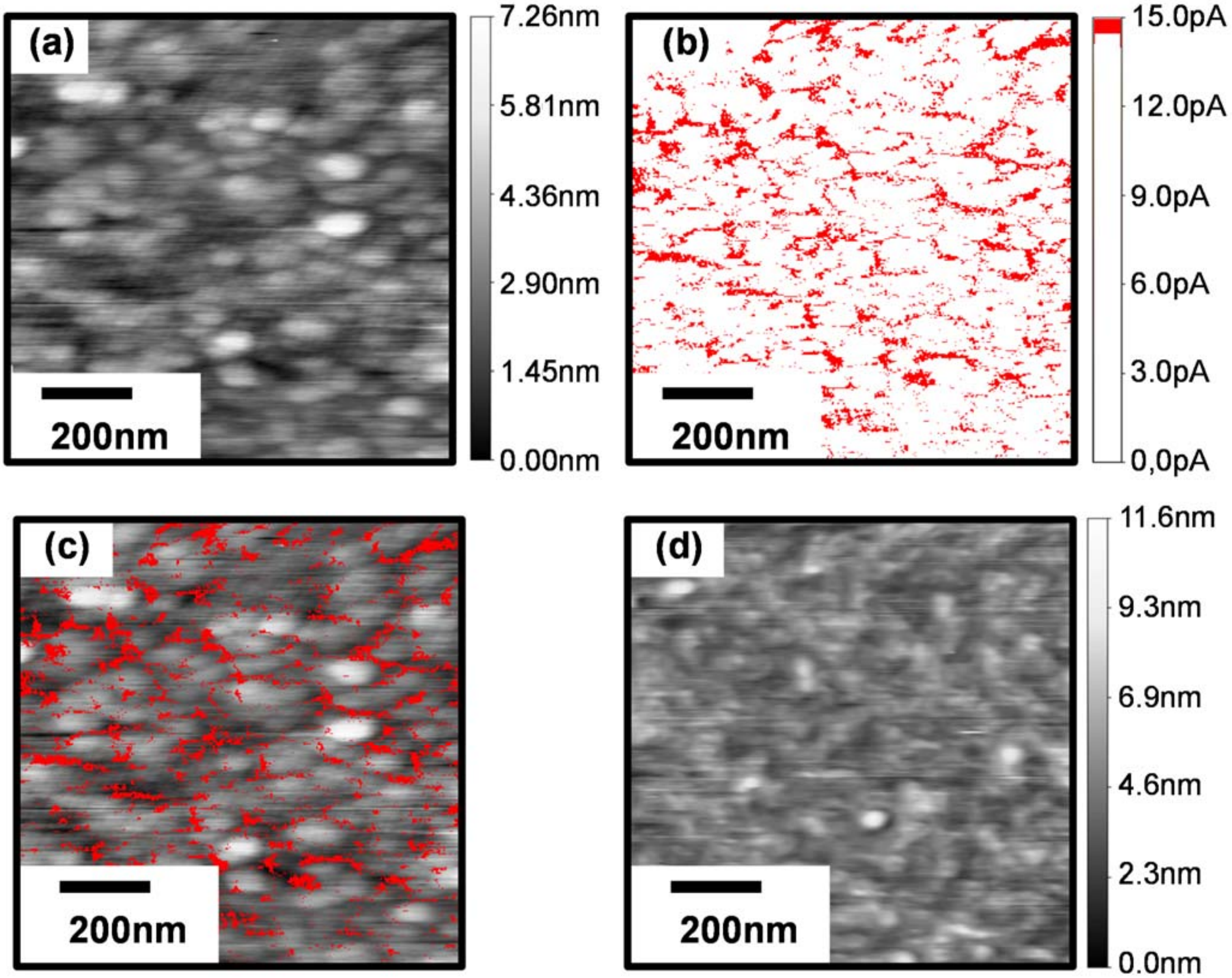

Figure S6. **(a)** The AFM topography image , **(b)** The CAFM current image (applied bias : 1V) of 3 nm Pt/ 3 nm TiN / 500°C annealed STO film on $Pt/TiO_2/SiO_2/Si$ substrate, and **(c)** the overlapped image of (a) and (b). **(d)** The AFM topography image of 500°C annealed STO film on $Pt/TiO_2/SiO_2/Si$ substrate.

Table S1. Base and possible Moiré patterns calculated from the spots of Figure S5

Figure S5	Base patterns				Moiré patterns			
	T_A [Å]	α_A [º]	T_B [Å]	α_B [º]	T_M [Å]	α_M [º]	T_m [Å]	α_m [º]
(a)	2.33	-48.4	1.79	57.7	1.26	89.0	1.66	14.5
	2.33	-48.4	1.75	14	1.88	59.6	1.16	-12.3
	2.33	-48.4	5.67	-3.1	3.03	-70.7	1.76	-35.6
	2.33	-48.4	1.84	77	1.15	-79.2	2.19	27.1
	2.33	-48.4	3.61	41.1	1.97	-81.4	1.95	-15.7
	2.33	-48.4	1.73	-84	2.95	48.5	1.04	-68.9
	2.33	-48.4	2.25	20.8	2.02	74.7	1.39	-13.1
	1.79	57.7	1.75	14	2.38	-52.5	0.95	35.6
	1.79	57.7	5.67	-3.1	2.01	75.7	1.51	44.3
	1.79	57.7	1.84	77	5.40	-18.0	0.92	67.2
	1.79	57.7	3.61	41.1	3.29	72.8	1.21	52.2
	1.79	57.7	1.73	-84	0.93	77.2	2.68	-16.0
	1.79	57.7	2.25	20.8	2.98	-69.6	1.05	41.4
	1.75	14.0	5.67	-3.1	2.46	21.3	1.35	10.0
	1.75	14.0	1.84	77	1.72	-42.2	1.05	44.6
	1.75	14.0	3.61	41.1	2.87	-7.2	1.21	22.8
	1.75	14.0	1.73	-84	1.15	55.3	1.33	-35.4
	1.75	14.0	2.25	20.8	7.13	-8.0	0.99	17.0
	5.67	-3.1	1.84	77	1.85	-84.3	1.67	60.2
	5.67	-3.1	3.61	41.1	5.14	80.3	2.37	24.2
	5.67	-3.1	1.73	-84	1.73	78.4	1.59	-68.0
	5.67	-3.1	2.25	20.8	3.42	35.0	1.64	14.1
	1.84	77.0	3.61	41.1	2.79	-76.0	1.27	65.1
	1.84	77.0	1.73	-84	0.90	86.8	5.31	-13.9
	1.84	77.0	2.25	20.8	2.11	-51.7	1.15	52.0
	3.61	41.1	1.73	-84	1.30	78.9	2.10	-55.6
	3.61	41.1	2.25	20.8	4.80	-6.7	1.41	28.6
	1.73	-84.0	2.25	20.8	1.23	64.1	1.58	-41.2
(b)	1.96	-39.4	2.74	-82.7	2.86	6.3	1.23	-57.3
	1.96	-39.4	1.96	52.9	1.36	-83.3	1.41	6.8
	1.96	-39.4	2.39	14.0	2.35	88.4	1.20	-15.5
	1.96	-39.4	2.78	7.9	2.67	-84.2	1.25	-20.1
	1.96	-39.4	2.76	-46.3	6.38	-23.3	1.15	-42.3
	1.96	-39.4	2.25	42.5	1.59	-83.9	1.38	-1.9
	2.74	-82.7	1.96	52.9	1.23	71.2	2.80	7.2
	2.74	-82.7	2.39	14.0	1.71	52.2	1.92	-30.0

	2.74	-82.7	2.78	7.9	1.94	53.0	1.96	-37.8
	2.74	-82.7	2.76	-46.3	4.40	26.1	1.45	-64.6
	2.74	-82.7	2.25	42.5	1.39	67.0	2.64	-9.4
	1.96	52.9	2.39	14.0	3.11	-72.2	1.14	35.4
	1.96	52.9	2.78	7.9	2.77	-82.3	1.24	34.5
	1.96	52.9	2.76	-46.3	1.49	85.1	1.73	14.6
	1.96	52.9	2.25	42.5	9.22	-79.4	1.05	48.1
	2.39	14.0	2.78	7.9	13.93	46.2	1.29	11.2
	2.39	14.0	2.76	-46.3	2.53	66.8	1.48	-13.8
	2.39	14.0	2.25	42.5	4.68	-68.5	1.20	28.7
	2.78	7.9	2.76	-46.3	3.04	71.2	1.56	-19.3
	2.78	7.9	2.25	42.5	3.96	-83.5	1.30	27.1
	2.76	-46.3	2.25	42.5	1.76	82.2	1.73	3.8
**(c)**	2.82	27.1	1.98	-15.8	2.91	-60.3	1.25	1.7
	2.82	27.1	2.83	-60.9	2.03	73.0	1.96	-16.8
	2.82	27.1	3.07	36.2	16.35	-30.3	1.47	31.5
	2.82	27.1	2.99	0.0	6.15	-83.4	1.49	14.0
	2.82	27.1	1.72	-31.8	2.00	-69.1	1.22	-10.1
	2.82	27.1	2.82	60.4	4.92	-46.3	1.47	43.8
	1.98	-15.8	2.83	-60.9	2.80	28.6	1.26	-34.2
	1.98	-15.8	3.07	36.2	2.51	-55.9	1.33	4.2
	1.98	-15.8	2.99	0.0	4.89	-42.2	1.20	-9.5
	1.98	-15.8	1.72	-31.8	5.92	-87.2	0.93	-24.4
	1.98	-15.8	2.82	60.4	1.84	-55.1	1.46	14.5
	2.83	-60.9	3.07	36.2	1.96	79.7	2.22	-15.0
	2.83	-60.9	2.99	0.0	2.87	62.2	1.69	-31.4
	2.83	-60.9	1.72	-31.8	3.10	0.4	1.10	-42.7
	2.83	-60.9	2.82	60.4	1.62	89.7	2.88	-0.1
	3.07	36.2	2.99	0.0	4.87	-69.6	1.59	17.9
	3.07	36.2	1.72	-31.8	1.82	-65.1	1.31	-8.6
	3.07	36.2	2.82	60.4	6.88	-52.9	1.50	48.8
	2.99	0.0	1.72	-31.8	2.89	-62.5	1.13	-20.3
	2.99	0.0	2.82	60.4	2.88	-62.7	1.68	31.2
	1.72	-31.8	2.82	60.4	1.44	-62.6	1.49	0.2
